# Catalytically Active Ti‐Based Nanomaterials for Hydroxyl Radical Mediated Clinical X‐Ray Enhancement

**DOI:** 10.1002/advs.202406198

**Published:** 2024-11-05

**Authors:** Lukas R. H. Gerken, Claire Beckers, Beatrice A. Brugger, Vera M. Kissling, Alexander Gogos, Shianlin Wee, Maria R. Lukatskaya, Hans Schiefer, Ludwig Plasswilm, Martin Pruschy, Inge K. Herrmann

**Affiliations:** ^1^ Nanoparticle Systems Engineering Laboratory Institute of Energy and Process Engineering (IEPE) Department of Mechanical and Process Engineering (D‐MAVT) ETH Zurich Sonneggstrasse 3 Zurich 8092 Switzerland; ^2^ Particles‐Biology Interactions Laboratory Department of Materials Meet Life Swiss Federal Laboratories for Materials Science and Technology (Empa) Lerchenfeldstrasse 5 St. Gallen 9014 Switzerland; ^3^ Laboratory for Applied Radiobiology Department of Radiation Oncology University Hospital Zurich University of Zurich Winterthurerstrasse 190 Zurich 8057 Switzerland; ^4^ Electrochemical Energy Systems Laboratory Institute of Energy and Process Engineering (IEPE) Department of Mechanical and Process Engineering (D‐MAVT) ETH Zurich Sonneggstrasse 3 Zurich 8092 Switzerland; ^5^ Department of Radiation Oncology Cantonal Hospital St. Gallen (KSSG) Rorschacherstrasse 95 St. Gallen CH‐9007 Switzerland; ^6^ Department of Radiation Oncology Inselspital University Hospital Bern 3010 Switzerland; ^7^ The Ingenuity Lab Balgrist University Hospital Forchstrasse 340 Zurich 8008 Switzerland; ^8^ Faculty of Medicine University of Zurich Rämistrasse 71 Zurich 8006 Switzerland

**Keywords:** photocatalyst, radiosensitization, radiotherapy, reactive oxygen species, titanium

## Abstract

Nanoparticle radioenhancement offers a promising strategy for augmenting radiotherapy by locally increasing radiation damage to tumor tissue. While past research has predominantly focused on nanomaterials with high atomic numbers, such as Au and HfO_2_, recent work has revealed that their radioenhancement efficacy decreases considerably when using clinically relevant megavoltage X‐rays as opposed to the orthovoltage X‐rays typically employed in research settings. Here, radiocatalytically active Ti‐based nanomaterials for clinical X‐ray therapy settings are designed. A range of candidate materials, including TiO_2_ (optionally decorated with Ag or Pt nanoseeds), Ti‐containing metal–organic frameworks (MOFs), and 2D Ti‐based carbides known as Ti_3_C_2_T*_x_* MXenes, is investigated. It is demonstrated that these titanium‐based candidates remain consistently performant across a wide energy spectrum, from orthovoltage to megavoltage. This sustained performance is attributed to the catalytic generation of reactive oxygen species, moving beyond the simple physical dose enhancements associated with photoelectric effects. Beyond titania, emergent materials like titanium‐based MOFs and MXenes exhibit encouraging results, achieving dose‐enhancement factors of up to three in human soft tissue sarcoma cells. Notably, these enhancements are absent in healthy human fibroblast cells under similar conditions of particle uptake, underscoring the selective impact of titanium‐based materials in augmenting radiotherapy across the clinically relevant spectral range.

## Graphical abstract

Ti‐based radiocatalytic nanomaterials, including MXenes, metal–organic frameworks and inorganic metal oxides, are explored for their potential to enhance X‐ray therapy and compared to traditionally used high‐Z nanoparticles. The radioenhancement efficacies are quantified based on metal uptake in fibrosarcoma cancer cells and mechanisms are studied using acellular 2′,7′‐dichlorofluorescein and in vitro DMSO radical quenching as well as DNA damage assays.

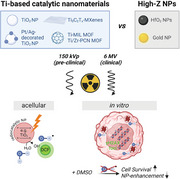

## 1 Introduction

Radiation therapy plays an important role in the fight against cancer.^[^ ^1^ ^]^ Technical advances of high‐energy photon dose delivery have contributed to tumor irradiation with highest conformity while limiting damage to normal tissue.^[^ ^2^ ^]^ Nevertheless, radiation resistance remains one of the leading challenges for successful radiotherapy,^[^ ^3^ ^]^ thus requiring additional advancements and innovations for improved treatment.

The use of radio‐enhancing substances, including small molecule drugs, and inorganic nanomaterials represents a promising strategy to improve tissue specificity of radiation treatment. HfO_2_ nanoparticles have been approved for clinical use in the EU for the treatment of locally advanced soft tissue sarcoma.^[^ ^4^ ^]^ At low energies (keV photons), nanoparticles containing high‐Z elements have been demonstrated to be highly effective in enhancing radiation damage by locally increasing X‐ray absorption and emission of secondary species.^[^ ^5^ ^]^ However, to be able to reach deep‐seated tumors, > 1 MV linear accelerators are used in clinics. At those energies (MeV photons), the photoelectric effect with its strong atomic number dependence becomes negligible and the (catalytic) activities of nanoparticles promoting the production of reactive oxygen species (ROS) gain in relevance for increasing radiation damage to cancerous cells.^[^ ^6^, ^7^ ^]^ Therefore, semiconductor nanoparticles promoting ROS generation via electron–hole pair formation, or transition metal‐based nanoparticles facilitating ROS generation via catalysis of the Haber–Weiss and Fenton reactions are promising candidate materials for high energy radiotherapy.^[^ ^6^ ^]^ In particular, TiO_2_ is known for its potent photocatalytic properties.^[^ ^8^, ^9^ ^]^ To further enhance charge separation, metal decoration on the surface of TiO_2_ has been exploited in photocatalysis as a promising strategy.^[^ ^10^, ^11^, ^12^, ^13^ ^]^ Alternatively, certain metal organic frameworks (MOFs) also exhibit promising photocatalytic activity paired with a high porosity and a large accessible surface area and have therefore been proposed for (visible) light induced ROS‐mediated cancer cell destruction.^[^ ^14^, ^15^, ^16^, ^17^ ^]^ Interestingly, in recent studies on Ti‐MIL‐125 and Ti/Zr‐PCN‐415, Ti‐based MOFs have been shown to outperform Hf‐based MOFs.^[^ ^18^, ^19^ ^]^ as well as HfO_2_ and TiO_2_ nanoparticles in in vitro radioenhancement using orthovoltage X‐rays.^[^ ^20^ ^]^ Similarly, 2D materials, especially Ti_3_C_2_T*_x_* MXenes, have also recently been suggested as potent radioenhancers.^[^ ^21^ ^]^ However, a significant limitation of these earlier studies is their exclusive use of low‐energy (keV) irradiation conditions, which does not allow for an informed selection of materials suitable for clinical X‐ray therapy. Our work aims to address this crucial gap, evaluating the potential of these novel materials under conditions that are clinically relevant. In this study, we conduct a comprehensive evaluation of Ti‐based candidate materials, including TiO_2_ (optionally with Ag or Pt), TiN, Ti‐MIL‐125 MOFs, Ti/Zr‐PCN‐415 MOFs, and Ti_3_C_2_T*_x_* MXenes, setting them against established benchmarks like HfO_2_ and Au in the context of radio‐enhancement. Our experiments span a range of irradiation conditions, from 150 kVp to the clinically relevant 6 MV photon irradiation. Following an in‐depth material characterization and quantification of ROS generation in cell‐free conditions, we provide insights into the radio‐enhancement mechanisms in human soft tissue sarcoma cells (HT1080) in direct comparison to noncancerous fibroblasts (NHDF) under clinical high energy photon irradiation conditions.

## 2 Results and Discussion

### 2.1 Nanomaterial Characterization: Composition, Size, Shape, and Crystallinity

**Figure 1:**
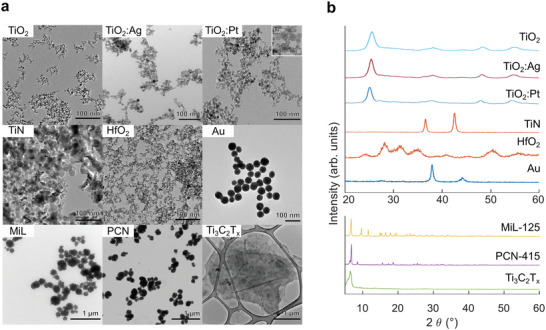
Nanomaterial characterization. TEM micrographs showing the nanomaterial morphology a). XRD patterns of nanomaterials showing peaks for material specific crystalline phases b).

We synthesized a set of Ti‐based nanomaterials and fully characterized morphology and crystal phase by transmission electron microscopy (TEM) and X‐ray diffraction (XRD) (**Figure** **1**). All nanomaterial samples exhibited a high degree of crystallinity. The materials examined in this study fall into three distinct categories, namely, i) inorganic nanoparticles (TiO_2_, Ag or Pt decorated TiO_2_ (TiO_2_:Ag, TiO_2_:Pt), TiN, HfO_2_, and Au), ii) metal organic frameworks (MIL‐125 and PCN‐415 MOFs), and iii) 2D materials (Ti_3_C_2_T*_x_* MXenes). All inorganic nanoparticles showed a spherical morphology with a size of below 100 nm (Table S1, Supporting Information). The TiN nanoparticles had a crystal size (*d*_XRD_ ± ESD) of 21.3 ± 0.3 nm based on the Rietveld refinement. The commercial Au nanoparticles (used as benchmark) exhibited a primary particle size *d*_TEM_ of 47 ± 6 nm (based on TEM, *n* = 102). The TiO_2_ nanoparticles featured a mean crystal size *d*_XRD_ of 5.7 ± 0.1 nm and a phase composition of ≈70% anatase and 30% rutile. Similarly, the Ag‐decorated TiO_2_ nanoparticles showed a mean crystal size of 6.6 ± 0.1 nm and a phase composition similar to the pure TiO_2_ nanoparticles. The presence of ≈1.6 ± 0.1 nm Ag nanoparticles was confirmed by the Rietveld refinement of the XRD pattern. For the Pt‐decorated TiO_2_ nanoparticles, a mean crystal size of 6.9 ± 0.1 nm was refined for TiO_2_ (with 64% anatase and 36% rutile phases) and 1.5 ± 0.2 nm for Pt nanoparticles, respectively. The presence of small (≈2 nm) Pt nanoparticles on the surface of the TiO_2_ nanoparticles was visible in TEM micrographs (Figure 1a inset) and also on high resolution scanning transmission electron microscopy (STEM) images (Figure S1, Supporting Information). The flame spray pyrolysis (FSP) synthesized HfO_2_ nanoparticles had a mean crystal size of 5.6 ± 0.1 nm and were composed of monoclinic (77%) and orthorhombic (23%) phases. All crystallite sizes were generally in good agreement with the primary particle sizes based on TEM (*d*_TEM_ of 18 ± 7 nm (TiN, *n* = 167), 6.1 ± 1.8 (TiO_2_, *n* = 125), 7.6 ± 2.4 nm (TiO_2_:Ag, *n* = 108), 7.2 ± 2.9 nm (TiO_2_:Pt, *n* = 112), and 5.4 ± 1.9 nm (HfO_2_, *n* = 114)). The MOF particles were larger than the inorganic nanoparticles, which is also reflected by sharper XRD peaks.

The MIL‐125 and PCN‐415 nanoparticles were sized around 200 nm (MIL‐125 diameter *d*_TEM_ of 252 ± 61 nm, *n* = 118; PCN diameter *d*_TEM_ of 180 ± 80 nm, *n* = 269), in agreement with previous in‐depth TEM analysis.^[^ ^20^ ^]^ The MXene flakes were distinctively different from the aforementioned nanoparticles in both size and morphology. The layered Ti_3_C_2_T*_x_* flakes had a lateral dimension of several microns (≈3 µm) and were only a few nanometers thick. The XRD pattern showed a characteristic (002) peak at 6.6° (2θ), which corresponds to an interlayer spacing of 1.34 nm (determined using the Bragg equation).^[^ ^22^, ^23^ ^]^ In summary, the primary particle sizes of the metal and metal oxide/nitride nanoparticles were in the range of 5–10 nm for the metal oxides (TiO_2_, TiO_2_:Ag/Pt, HfO_2_), around 20 nm for TiN and around 50 nm for Au nanoparticles. The MOF particles MIL‐125 and PCN‐415 were around 100–300 nm in size, while the Ti_3_C_2_T*_x_* MXene flakes had a 2D sheet‐like structure with interplanar spacings of 1.34 nm and flake diameters of several micrometers.

All nanomaterials showed a negative Zeta‐potential between −25 and −55 mV and a rather polydisperse (with PDIs > 0.3) and agglomerated water dispersion behavior with hydrodynamic sizes larger than their primary particle sizes (Table S2, Supporting Information). An exception was observed with citrate stabilized Au nanoparticles, which revealed a hydrodynamic size in water close to the primary particle size, indicating monodisperse (PDI < 0.2) behavior. Since dynamic light scattering (DLS) results reflect a light scattering intensity‐weighted average value, rather than a real size distribution, it is known that z‐Averages of polydisperse samples are strongly overestimated and biased toward the largest particle fractions, as scattered light is inversely proportional to the sixth power of the nanoparticle radius.^[^ ^24^ ^]^ However, a general trend was still observed for all nanomaterials: PBS tended to further agglomerate nanomaterial suspensions, while 10% FCS supplemented cell medium indicated stabilization of nanomaterials, with hydrodynamic sizes similar or even smaller to those in water. The proteins in FCS form a complex corona around nanoparticles that can influence nanomaterial‐cell interactions, cellular uptake and toxicity.^[^ ^25^, ^26^, ^27^, ^28^ ^]^ Additionally, we have previously shown how colloidal nanomaterial stability can affect sedimentation behavior, cellular nanomaterial uptake and toxicity in in vitro setting.^[^ ^5^ ^]^ Therefore, we emphasize the necessity of documenting nanomaterial uptake (e.g., quantified via ICP‐OES and visually confirmed by TEM) in addition to the nominal nanomaterial concentration in cellular assays.

### 2.2 Nanomaterial‐Induced Reactive Oxygen Species Formation as a Function of X‐Ray Energy

**Figure 2:**
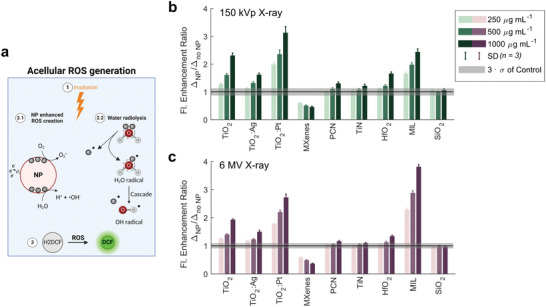
Schematic of acellular ROS enhancement quantification using the H_2_DCF‐DA assay a). Nanoparticle (NP) ROS enhancement (displayed as a change in fluorescence before and after irradiation of nanoparticle fluorophore solutions compared to the fluorescence change for nanoparticle‐free fluorophore solutions) after 12 Gy 150 kVp X‐ray b) or 12 Gy 6 MV X‐ray c) irradiation. Nanoparticles were tested at three different mass concentrations. Bars and error bars indicate the mean and SD of *n* = 3 technical replicates. The shaded gray area indicates 3 SDs from 3 independent control experiments (no nanoparticles). Δ: change in fluorescence before and after 12 Gy X‐ray irradiation of a 50% PBS solution with the H_2_DCF fluorophore (with or without nanoparticles). The ROS enhancement of Au is not displayed due to a severe background interaction of nanoparticles with the fluorophore (0 Gy fluorescence signal with Au NPs > 3.5 × 10^6^). SiO_2_ (A380) was added as a negative nanoparticle control. Data exemplarily shown for one experiment. Reproducibility was shown for 150 kVp irradiation (Figure S4, Supporting Information).

After physicochemical characterization, we initially quantified the ROS generation capability of various Ti‐based materials in a cell‐free setting (**Figure** **2**a). The levels of free ROS generated by X‐ray interactions with nanoparticles and with water molecules (water radiolysis) were determined by the use of the fluorescent 2′,7′‐dichlorofluorescein (DCF) indicator. Notably, DCF is a widely recognized, nonspecific ROS probe that is, nevertheless, highly reactive toward hydroxyl radicals (∙OH).^[^ ^29^ ^]^ The measurements were conducted in a 50% PBS dispersant, both with and without nanomaterials, after being irradiated with 12 Gy from either a 150 kVp or 6 MV X‐ray source (Figure 2b,c; and Figures S2 and S3, Supporting Information). The ROS enhancement factor — defined as the ratio of X‐ray‐induced fluorescence change with and without nanomaterials — served as a key metric. A factor greater than 1 suggests additional ROS generation, while a factor lower than 1 indicates ROS scavenging. TiO_2_‐based nanoparticles and MIL‐125 MOFs consistently demonstrated the highest ROS enhancement factors. HfO_2_ nanoparticles also showed an increase in ROS levels, albeit to a lesser extent, in proportion to their concentration. Conversely, PCN‐415, TiN, and the control SiO_2_ nanoparticles exhibited baseline ROS levels postirradiation. The MXene flakes showed a below baseline level fluorescence, hinting at a possible ROS quenching mechanism,^[^ ^30^ ^]^ as assay interference was nonobvious (unaltered baseline). Gold nanoparticles were excluded due to their propensity to oxidize the fluorophore, causing high background fluorescence. The effectiveness of ROS enhancement under 150 kVp X‐ray irradiation was, thus, in the following order: TiO_2_:Pt > MIL‐125 > TiO_2_ > HfO_2_, TiO_2_:Ag > PCN‐415, TiN, SiO_2_ ≈1 > MXenes.

To directly assess the influence of the photon energy, nanomaterial ROS enhancement was measured in 6 MV photon irradiated samples using otherwise identical conditions. Again, the highest ROS enhancement factors were found for all TiO_2_‐based nanoparticles and the MIL‐125 MOFs. HfO_2_ nanoparticles also showed slightly increased ROS levels with increasing nanoparticle concentration, while PCN‐415, TiN, and the SiO_2_ again showed baseline level ROS concentrations after irradiation. The effectiveness of ROS enhancement of the different nanomaterials under clinically relevant 6 MV X‐ray irradiation conditions was, thus, in the following order: MIL‐125 > TiO_2_:Pt > TiO_2_ > TiO_2_:Ag > HfO_2_ > PCN‐415, TiN, SiO_2_ ≈1 > MXenes. Notably, both the magnitude of ROS generation and the performance of the different Ti‐based nanomaterials was highly similar between kVp and MV X‐ray irradiation setups. This can be explained by the underlying catalytic mechanism, in contrast to high‐Z materials (e.g., Au and HfO_2_), where a strong photon energy dependence is found,^[^ ^31^ ^]^ and which generate ROS primarily via a source‐energy‐dependent photoelectron‐based mechanism. For semiconductor materials, a catalytic ROS production mechanism after excitation with photons is relatively established.^[^ ^32^ ^]^ Irradiation induced electrons and holes are available for oxidative and reductive water splitting reactions on the surface of the photocatalyst. Since the number of electron–hole pairs generated in a semiconductor strongly depends strongly on the energy deposited inside the nanoparticle and the semiconductor's bandgap energy,^[^ ^33^, ^34^ ^]^ it is generally expected that the photocatalytic ROS production activity is higher for preclinical (kVp) than for clinical (MV) X‐ray sources. A previous Monte Carlo simulation has demonstrated this by calculating the energy deposition in scintillating nanoparticles and the photodynamic singlet oxygen generation in a tumor‐like environment using 100 and 500 keV photons.^[^ ^35^ ^]^ However, the study also showed that for some of the materials the energy fraction deposited in the nanoparticles within the tumor volume even increased with the higher energetic photons, suggesting a less direct dependence of on the energy of incident photons on singlet oxygen generation. Analogous to this, for radiocatalytic ROS generation, in addition to energy absorption by nanoparticles, also charge migration, recombination, and hydroxyl radical conversion efficacy should be considered. While TiO_2_ has a positive valence band potential sufficient for producing ▪OH radicals, Pt on the surface of TiO_2_ acted as effective cocatalyst promoting charge transfer due to the metal‐semiconductor junction, suppressing electron–hole pair recombination and boosting the radiocatalytic ROS generation of TiO_2_.^[^ ^9^, ^10^, ^36^, ^37^ ^]^ In a direct comparison, Ag was a less efficient cocatalyst than Pt, in line with a higher Schottky barrier,^[^ ^38^ ^]^ and TiO_2_:Ag produced less ROS than TiO_2_ due to a higher primary particle size and, therefore, lower accessible surface area. TiN as a low‐Z nanoparticle with similar physical X‐ray interaction properties compared to TiO_2_ showed almost no ROS generation, highlighting the importance of valence and conduction energy band configuration for effective radiocatalytic ▪OH production. In line with this, amorphous low‐Z SiO_2_ nanoparticles acted as a negative control particle for the assay. The strong photon induced ROS generation ability of MIL‐125 MOFs may be based on photoinduced charge separation.^[^ ^14^ ^]^ For PCN‐415 MOFs, ROS generation was low, which may be caused by phosphate incorporation into the MOF.^[^ ^39^ ^]^

### 2.3 Nanomaterial Cytocompatibilty, Cellular Uptake, and Intracellular Distribution

**Figure 3:**
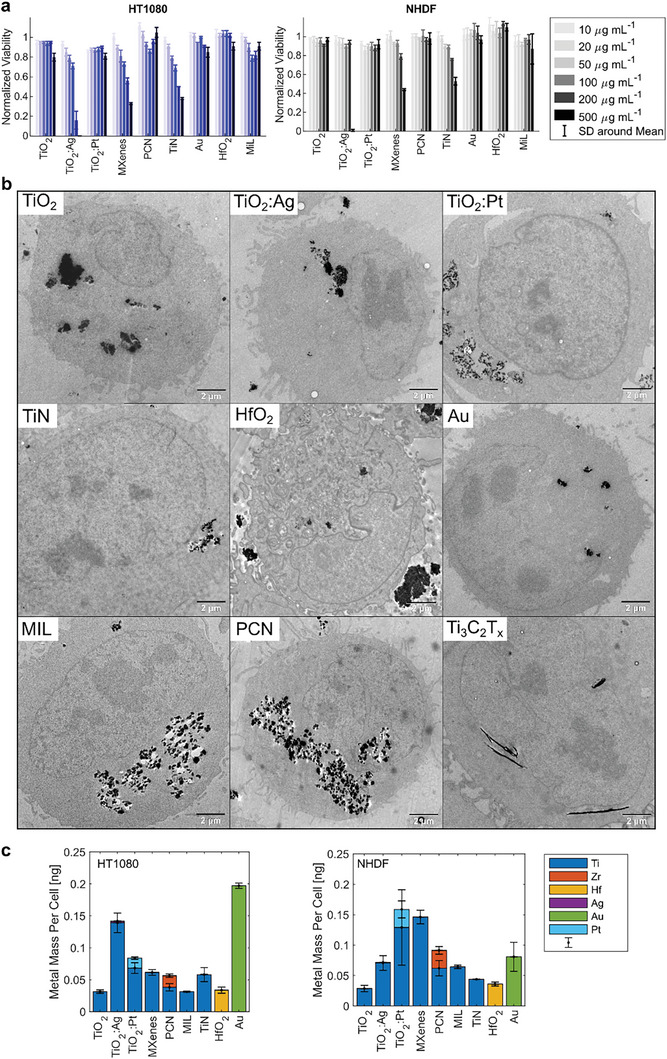
Viability of HT1080 and NHDF cells after 24 h nanoparticle incubation at different concentrations normalized to the viability of control cells. Bars and error bars represent the mean and SD (*n* = 4) a); TEM micrographs of nanomaterials in HT1080 cells b) and corresponding ICP‐OES uptake quantification (in metal mass per cell) of nanomaterials in cancerous HT1080 and noncancerous NHDF cells c). Before harvesting for analysis, cells were incubated for 24 h with either 100 µg mL^−1^ (all metal oxides, TiN, or Au nanoparticles), 50 µg mL^−1^ (MIL‐125 or PCN‐415 MOFs) or 20 µg mL^−1^ (Ti_3_C_2_T*_x_* MXenes) of nanomaterial containing cell medium solutions. TEM scale bars: 2 µm. Bars and error bars in (c) indicate the mean and standard error of the mean (SEM, *N* = 3), respectively.

Prior to radioenhancement experiments with nanomaterials in cell cultures, we assessed the cell compatibility of all nanoparticles using a metabolic activity‐based cell viability assay. Human fibrosarcoma (HT1080) and normal human dermal fibroblast (NHDF) cells were exposed to increasing concentrations of the nanomaterials for 24 h. Among the materials investigated, only a few — specifically TiO_2_:Ag, MXenes, and TiN — exhibited significant decreases in cell viability at concentrations exceeding 50 µg mL^−1^ in HT1080 cells (**Figure** **3**). All other Ti‐materials were well tolerated in absence of irradiation. Overall, NHDF cells demonstrated even better tolerance to the nanomaterials compared to HT1080 cells. The most notable toxicity was observed with Ag‐containing TiO_2_. The primary mechanisms of toxicity for Ag nanoparticles have previously been linked to the release of Ag^+^ ions, elevated oxidative stress, mitochondrial dysfunction, increased DNA damage, augmented apoptosis, and alterations in protein and gene expression.^[^ ^40^, ^41^, ^42^ ^]^

As uptake and intracellular distribution strongly influence radioenhancement, TEM and elemental analysis were performed. Transmission electron micrographs of HT1080 cells show that all nanomaterials were taken up by cells and formed intracellular agglomerates (Figure 3). As previously described,^[^ ^43^, ^44^ ^]^ the nanomaterials were likely internalized via endocytic pathways and were present as agglomerates of various sizes within the cytoplasmic compartments. No nanomaterials were detected inside the nucleus. The Ti_3_C_2_T*_x_* MXene sheets showed distinctive differences compared to the spherical inorganic and metal–organic nanoparticles. Intracellular sheets of several hundred nanometers to several micrometers in lateral length were detected with flake thicknesses of around 50 nm. The layered MXene flakes were found to be individually distributed in the perinuclear region. The corresponding metal uptake per HT1080 and NHDF cell was quantified using inductively coupled plasma optical emission spectrometry (ICP‐OES). Quantitative analysis showed that the Ti metal mass uptake in HT1080 cells was between 0.03 and 0.07 ng per cell for all Ti‐based nanomaterials, except for TiO_2_:Ag, which showed a considerably higher nanoparticle uptake with 0.15 ng per cell.

In NHDF cells, Ti metal mass uptake was similar, with around 0.03–0.07 ng per cell, however, for TiO_2_:Pt and MXenes, the uptake was higher and around 0.15 ng per cell. The Hf metal mass uptake per cell was around 0.03 ng per cell in both cell lines, while the Au mass uptake was around 0.2 ng per cell for HT1080 and 0.08 ng per cell in NHDF cells. In the case of the mixed metal materials, ICP‐OES confirmed molar metal ratios of 0.8 mol% for Ag:Ti, 5.3 mol% for Pt:Ti and 25 mol% for Zr:Ti, which are in agreement with the theoretical values of 1.25 mol% (in TiO_2_:Ag), 4.5 mol% (in TiO_2_:Pt), and 25 mol% (in PCN‐415), respectively (Figure S5, Supporting Information). A general trend was observed in that the low‐density materials MXenes and MOFs showed Ti mass uptakes comparable to those for the smaller sized oxide nanoparticles at lower nominal nanomaterial incubation concentration.

### 2.4 Nanomaterial Radioenhancement in Human Sarcoma Cells and Healthy Fibroblasts

**Figure 4:**
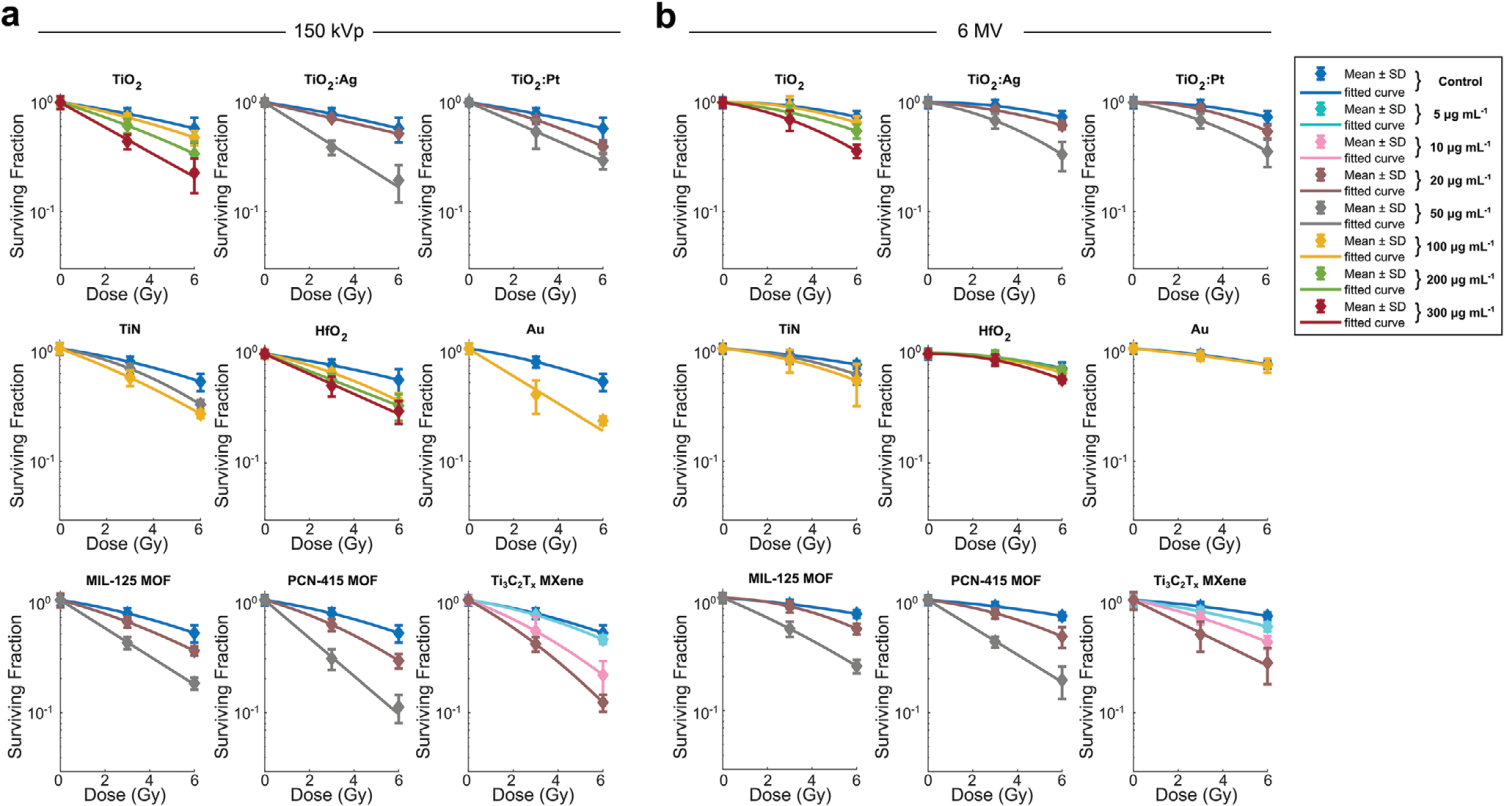
Survival curves (linear quadratic fits) of HT1080 cells incubated with different nanoparticles and concentrations, and irradiated with 0, 3 and 6 Gy of 150 kVp a) or 6 MV b) X‐rays. The surviving fractions were based on the metabolic activity of the cells 5 days postirradiation. Data expressed as average (mean ± SD, *n* = 6) from *N* = 2 independent biological experiments and normalized to the metabolic activity at 0 Gy for each nanoparticle condition.

Radioenhancement properties upon photon irradiation in cancer cells were investigated using a 150 kVp X‐ray tube source or a clinical 6 MV linear accelerator (**Figure** **4**). After 24 h of nanoparticle incubation, cells were washed and irradiated with 0, 3 and 6 Gy of X‐rays. The surviving fraction of HT1080 cells was quantified with a metabolic assay 5 days after irradiation. All cells with taken up nanomaterials showed a concentration dependent faster decline of the cell survival curve compared to nanomaterial‐free control cells, indicating more efficient cancer cell killing. The 50 nm sized Au nanoparticles served as benchmark for the radioenhancement with 150 kVp X‐rays, since they have a well‐known strong enhancement effect with those photons stemming from physical dose enhancement, that, however, vanishes at 6 MV X‐ray irradiation due to the absence of photoelectrons at megavoltage energies.^[^ ^31^ ^]^ All radioenhancer candidate nanomaterials were investigated exclusively at sub‐toxic doses. For HfO_2_ and TiO_2_, doses up to 300 µg mL^−1^ had no detectable influence on cell viability in absence of irradiation. However, since some of the other nanomaterials showed effects on cell viability even in the absence of irradiation at concentrations of 100 µg mL^−1^ (Figure S6, Supporting Information), MOF and metal‐decorated TiO_2_ nanomaterial concentrations were only investigated using subtoxic concentrations of 50 µg mL^−1^ or lower. For MXenes, doses up to 20 µg mL^−1^ were investigated.

**Figure 5:**
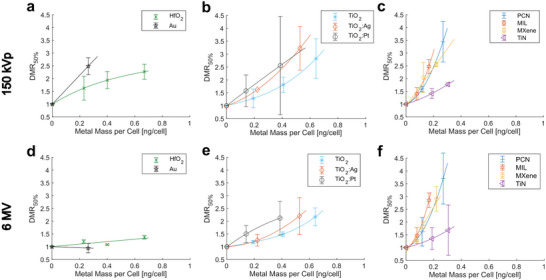
Magnitude of the radioenhancing effect of all nanomaterials for 150 kVp a–c) and 6 MV d,e) X‐ray treatment given as dose modifying ratios (DMR_50%_). The DMR_50%_ is the ratio of the lethal X‐ray dose (LD_50_) leading to 50% cell survival with nanoparticles compared to that without nanoparticles; it is plotted against the metal mass per cell (*x*‐axis) as quantified using ICP‐OES. Data given as mean ± SD from 2 independent biological experiments.

From cell viability curves displayed in **Figure** **5**, a magnitude of the nanomaterial radioenhancing effect can be calculated. We extracted the enhancement effect as the dose modifying ratio (DMR_50%_), which indicates the ratio by which the X‐ray dose can be reduced, leading to the same biological effect of a 50% cell survival when comparing two conditions. In our case, the DMR_50%_ is given as

(1) $$\mathrm{DM}R_{50\%}=\frac{{\mathrm{LD}}_{50\;\left(\mathrm{without}\;\mathrm{NPs}\right)}}{{\mathrm{LD}}_{50\;\left(\mathrm{with}\;\mathrm{NPs}\right)}}$$

where LD_50_ is the lethal X‐ray dose leading to 50% cell viability. Radioenhancement factors for all nanomaterials and for the two different X‐ray sources are summarized in Figure 5 and plotted against the metal mass per cell as quantified using ICP‐OES (Figure S7, Supporting Information). This allows comparison of the radioenhancement effect to the de facto present nanomaterial amount during irradiation, and thus enables to draw a comparison between nanomaterials. Overall, it is evident that the X‐ray energy had a major effect on the radioenhancement performance of the high‐Z materials, Au and HfO_2_ (Figure 5a,d).

The DMR of Au nanoparticles was 2.5 ± 0.3 under 150 kVp X‐ray irradiation, while it was around 1 (no enhancement) for the clinically more relevant 6 MV X‐ray irradiation. This drastic difference can be explained by the difference in the physical dose enhancement (i.e., secondary species ejection from the nanoparticle), which is high for low energy kV X‐rays and negligible for high energy MeV X‐rays.^[^ ^31^, ^45^ ^]^ For HfO_2_ nanoparticles, the highest DMRs were found to be 2.3 ± 0.3 and 1.4 ± 0.1 for kV and MV irradiation, respectively. Compared with the same mass uptake, HfO_2_ nanoparticles were not as effective as Au nanoparticles with kV X‐rays. However, there is a non‐negligible enhancement observed for HfO_2_ nanoparticles with MV irradiation, which has to stem from other than physical effects and could originate from catalytic ROS production (i.e., chemical enhancement), as further supported by the H_2_DCF‐DA‐based ROS measurements following 150 kVp and 6 MV irradiation (Figure 2).

Most importantly, Ti‐based materials retained their high radioenhancement properties even when using MV irradiations. TiO_2_ and metal‐decorated TiO_2_ nanoparticles showed mean DMRs around 2.5–3.2 for kV irradiation (Figure 5b) for the highest nanoparticle concentration, while they were around 2.2 for MV irradiation (Figure 5e). Physical enhancement effects can be neglected due to the low‐Z nature of TiO_2_.^[^ ^31^ ^]^ At the same metal mass uptake, Ag and Pt decoration slightly improved the radioenhancement effects of TiO_2_ nanoparticles. In case of Pt decoration, this improvement might be attributed to improved ROS generation as documented by our ROS quantification assay (Figure 2). In case of Ag decoration, this improvement likely stems from biological radiosensitization effects, such as mitochondrial dysfunction, increased oxidative or endoplasmic reticulum stress, or autophagy modulation, which can be triggered by Ag nanoparticles and by released Ag^+^ ions.^[^ ^46^, ^47^ ^]^

X‐ray energy‐independent radioenhancement effects were also observed for the Ti‐MOFs and MXenes. Mean DMRs around 3 were observed for highest concentrations of MOFs and MXenes, while they were below 2 for TiN nanoparticles with comparable metal mass uptake (**Figure** 5c,). As we did not find additional ROS generation with TiN nanoparticles under X‐ray irradiation, those particles might reduce the cellular antioxidant capacity and by this sensitize cancer cells to ionizing radiation.^[^ ^31^, ^48^ ^]^ MOFs and MXenes show radioenhancement properties comparable to those of Au NPs with kV X‐ray irradiation, which is, unlike for Au NPs, even retained with MV X‐ray irradiation. Here, we show that also Ti‐MOFs and Ti‐MXenes outcompete Au, HfO_2_, TiO_2_, and TiN nanoparticles in radioenhancement efficiency at the same cellular metal mass uptake, likely due to higher accessible surface area^[^ ^18^ ^]^ and hydroxyl radical creation.

### 2.5 Mechanistic Insights into Ti‐Based Radioenhancement

**Figure 6:**
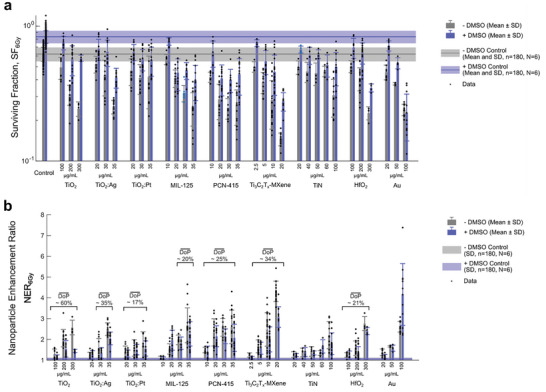
Effect of DMSO (a ∙OH radical quencher) during irradiation on the cell surviving fraction (SF) of HT1080 cells, pre‐treated with or without nanomaterials, 5 days after 6 Gy treatment with 150 kVp X‐rays a); nanomaterial enhancement ratio (NER_6Gy_) for the 6 Gy irradiation dose with and without DMSO protection b); the $\bar{\mathrm{DoP}}$ (Mean Degree of Protection (from DMSO) of the nanomaterial enhancement effect. The cell surviving fraction was determined via a metabolic assay.

To investigate ROS generation (chemical enhancement) as the putative key driver of nanoparticle radioenhancement, we applied the **∙**OH radical quencher DMSO during irradiation of HT1080 cells in vitro. For control cells (no nanomaterials), DMSO increased cell viability in response to 6 Gy irradiation from 62% (no DMSO) to 83% (with DMSO) (**Figure** **6**a), resulting in a protection effect of around 75%, which is well in line with literature reporting the contribution of indirect (ROS‐mediated) X‐ray radiation damage amounting to 63%–85%.^[^ ^49^, ^50^, ^51^, ^52^, ^53^ ^]^ For nanomaterial‐treated cells, nanoparticle enhancement ratios at 6 Gy X‐ray doses (NER_6Gy_) were calculated for conditions with and without DMSO. In nanomaterial‐treated cells, the DMSO protection effect greatly depends on the nanomaterial type. Figure 6b shows NER_6Gy_ values for each nanoparticle type and concentration comparing with or without DMSO presence during irradiation. The NERs compare the surviving fraction of control cells to that of cells with nanomaterials and are > 1 in case of X‐ray dose enhancement effects.

DMSO had the highest protection effects for TiO_2_ nanoparticles ($\bar{DoP}\;\approx \;60\%)$, followed by Ti_3_C_2_T*_x_* MXenes and TiO_2_:Ag nanomaterials ($\bar{DoP}\;\approx 35\%)$, followed by both MOF materials, HfO_2_ and TiO_2_:Pt nanomaterials ($\bar{DoP}\;\approx \;20\%)$. These data suggest, that in line with their ability to produce ROS during irradiation in PBS solutions, those materials also enhanced the production of cytotoxic hydroxyl radicals intracellularly during irradiation, which led to an increased cell killing effect. Nanomaterial TiN only showed DMSO quenching effects similar to control cells and thus, no relevant NER_6Gy_ quenching effects. Au nanoparticles also showed similar DMSO quenching effects than the control cells. Therefore, the NER_6Gy_ with DMSO was similar to the NER_6Gy_ without DMSO during irradiation for Au NPs. Taken together, these data show for all Ti‐based nanomaterials that an increased generation of **∙**OH radicals is one of the major mechanisms of nanomaterial enhanced radiotherapy.

**Figure 7:**
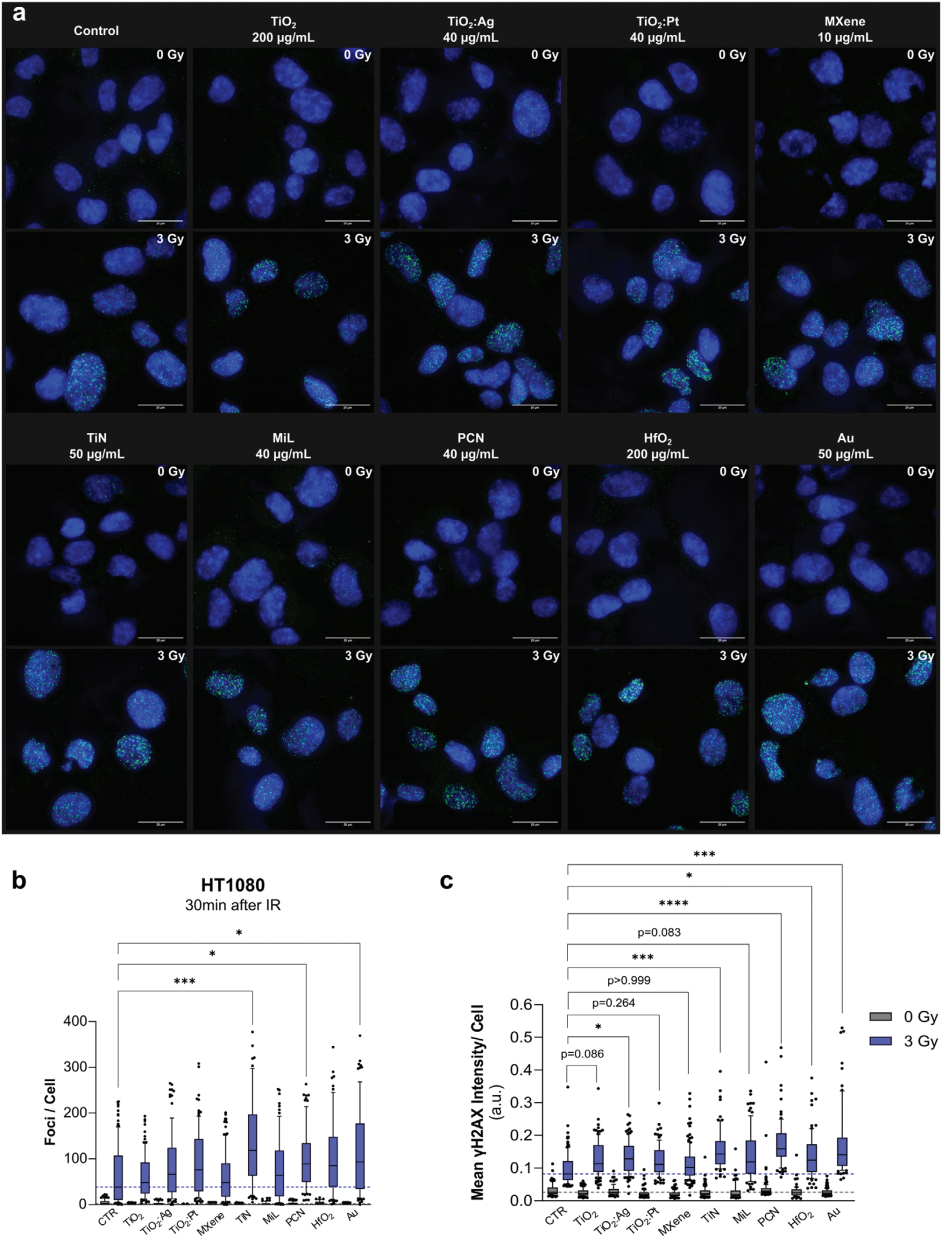
Representative immunofluorescence images showing γH2AX foci (green) in nuclei (blue) of HT1080 cells with and without nanomaterials 30 min after 0 or 3 Gy irradiation with 150 kVp X‐rays a); quantification of foci per nucleus b) and mean γH2AX intensity per nucleus c) for 0 and 3 Gy irradiated cells with and without nanomaterials; scale bars: 20 µm; the whiskers indicate 10th and 90th percentiles; data points below and above the whiskers are drawn as individual points; boxes extend from the 25th to 75th percentiles; number of analyzed nuclei was around 80 per 0 Gy and around 110 per 3 Gy condition; statistical significance was assessed with the Kruskal–Wallis and Dunn's multiple comparison test (*α*  =  0.05); asterisks indicate significance levels as: ****: *p* < 0.0001, ***: *p* < 0.001, **: *p* < 0.01, *: *p* < 0.05.

Radiation‐induced ROS are well known to induce double‐strand DNA breaks (DSBs), which are considered the most lethal form of DNA damage.^[^ ^54^ ^]^ Immunofluorescent staining of γH2AX foci was used to evaluate the nanomaterial radioenhancement effect on the generation of DSBs.^[^ ^55^ ^]^ Initial DSBs were assessed 30 min following radiation exposure. In addition, residual γH2AX foci 24 h postradiation exposure were measured to estimate the DNA damage repair capacities (via nonhomologous end‐joining or homology directed repair pathways^[^ ^54^, ^56^ ^]^) of cells treated with or without nanomaterials. At the early time point, 30 min after 0 or 3 Gy exposure to 150 kVp X‐rays, γH2AX foci number and intensity per nucleus assessed by immunofluorescence was increased in irradiated versus nonirradiated cells (**Figure** **7**). The median in the control population increased from 2 foci to 41 foci per cell for nonirradiated and irradiated cells, respectively. Without irradiation none of the nanomaterials led to increased DNA damage compared to control cells. With irradiation, however, more γH2AX foci and higher mean γH2AX intensity per cell was observed for all nanomaterials compared to irradiated control cells. Thus, an increase of DSBs in response to the combined treatment was consistent with previously determined dose enhancement effects at corresponding nanomaterial concentrations. At the 24 h postirradiation time point, we observed that most of the DNA damage was repaired. The residual number of foci per cell and the mean γH2AX intensity per cell was drastically reduced (Figure S8, Supporting Information) compared to the earlier time point. The residual median of the 3 Gy irradiated control cells dropped to around 6 foci per cell. Likewise, the residual DNA damage in nanomaterial treated cells was comparable to control cell levels 24 h after irradiation though with trends pointing toward higher residual DSBs for nanomaterial treated and irradiated cells, especially for those treated with TiO_2_, TiO_2_:Ag, TiO_2_:Pt, and Au.

Our data indicate that combined nanomaterial treatment and irradiation results in more DNA DSBs at 30 min and—albeit to a lesser extent—at 24 h post‐treatment compared to irradiated cells alone. This effect is in accordance with the data from our radiosensitization effect assays. Previous reports show radiosensitization and DSB damage induction results for Au nanoparticles in HeLa,^[^ ^57^, ^58^ ^]^ FaDu,^[^ ^59^ ^]^ SCCVII,^[^ ^60^ ^]^ Hs‐895‐SK,^[^ ^58^ ^]^ (hypoxic) EMT‐6,^[^ ^61^, ^62^ ^]^ and MDA‐MB‐231 cells,^[^ ^63^ ^]^ that are consistent with our findings. Increased DNA damage with irradiation and nanomaterials in different cell lines has been reported mostly for high‐Z nanomaterials, such as HfO_2_ nanoparticles (NBTXR3),^[^ ^64^ ^]^ Hafnium‐related MOFs,^[^ ^18^, ^65^, ^66^, ^67^ ^]^ CuO nanoparticles,^[^ ^68^ ^]^ AGuIX‐Bi nanoparticles,^[^ ^69^ ^]^ or WS_2_:Gd^3+^‐PEG nanoflakes.^[^ ^70^, ^71^ ^]^ Mechanistically, high‐Z nanoparticles (such as Au and HfO_2_) produce photoelectrons during 150 kVp X‐ray exposure that increase the energy deposition inside the cell's nucleus.^[^ ^31^ ^]^ Therefore, increased DNA damage can be attributed to physical interactions and released secondary species during keV X‐ray interactions with high‐Z nanomaterials. This physical dose enhancement for Au and HfO_2_ nanoparticles is strongly decreased during 6 MV X‐ray irradiation. This is reflected by markedly diminished DMR_50%_ values and DSB damage (Figure S9, Supporting Information) compared to 150 kVp X‐ray irradiation. The photoelectric cross‐section is very low for incident MeV photons and also for low‐Z nanoparticles (such as Ti‐based nanomaterials). The latter do not increase the physical dose inside a cell's nucleus during X‐ray irradiation but instead increase ROS, as indicated by our acellular H_2_DCFDA assay and in vitro DMSO protection assay. We thus conclude that ROS generation contributed to the increased amount of DSBs observed with Ti‐based radiocatalytic nanomaterials under preclinical and clinical irradiation conditions. We would like to point out that other biological mechanisms could also or additionally be possible drivers of enhanced DNA damage after irradiation. Nano‐TiN for example has been shown to downregulate the expression of antioxidant genes and reduce the antioxidant capacity in zebrafish embryos.^[^ ^48^ ^]^ In cancer cells, such a mechanism could potentially lead to more ROS and DNA damage after irradiation.

### 2.6 Absence of Radioenhancement in Healthy Fibroblasts Indicates Promising Therapeutic Ratio

**Figure 8:**
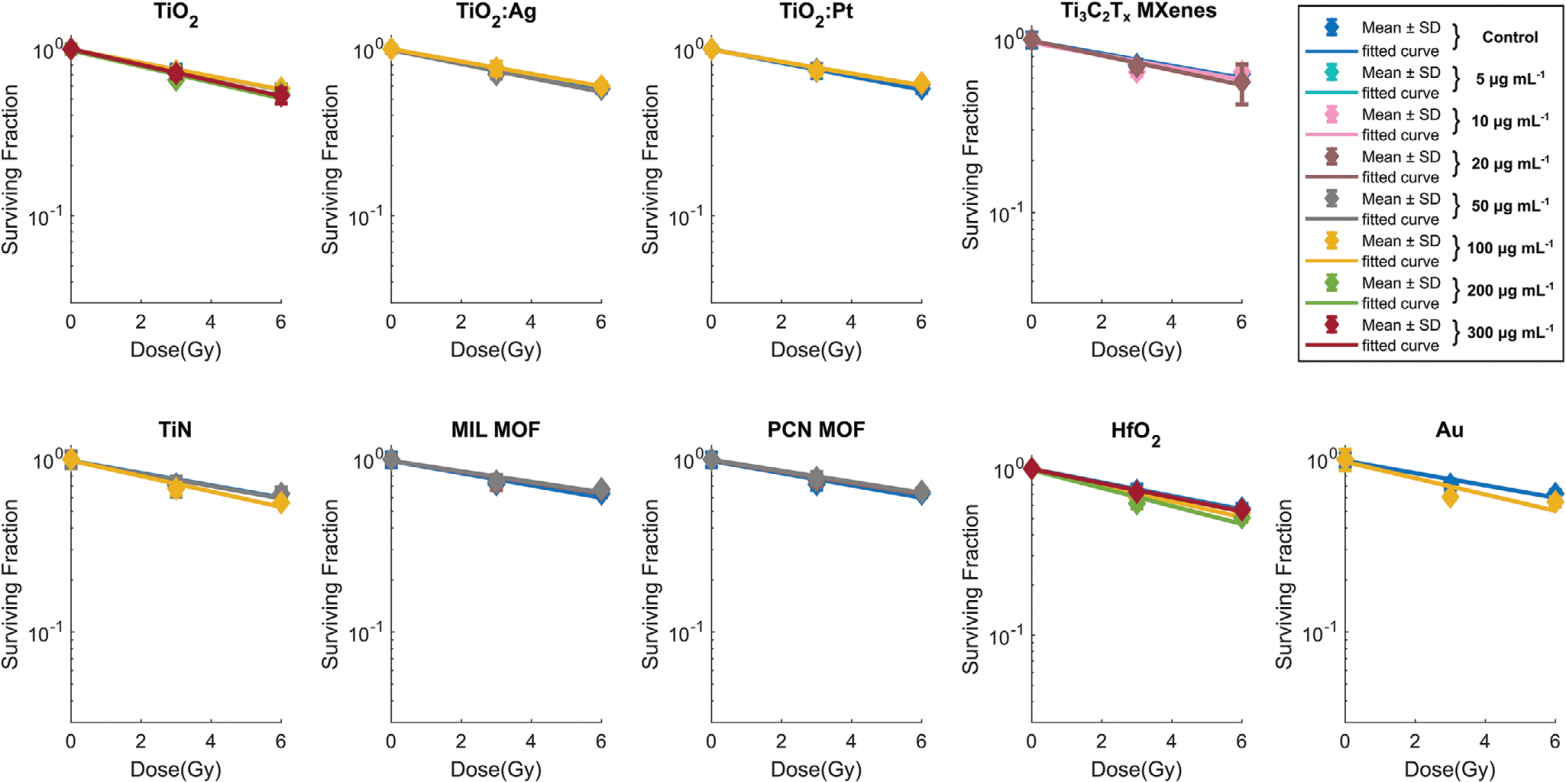
Survival curves (linear quadratic fit) of noncancerous NHDF cells incubated with different nanomaterials and concentrations, and irradiated with 0, 3, and 6 Gy of 6 MV X‐rays. The surviving fractions were based on the metabolic activity of the cells 7 days post irradiation. Data (mean ± SD, *n* = 3) was normalized to the metabolic activity at 0 Gy for each nanomaterial condition; one biological replicate (cell line).

Finally, the radioenhancing potential of all nanomaterials was quantified in noncancerous NHDF cells with clinical 6 MV X‐ray irradiation (**Figure** **8**). Notably, no significant radioenhancement effects were observed in NHDF cells for any nanomaterial, even though the nanomaterials were internalized by the cells to a similar extent as for the sarcoma cells (Figure 3). For the lower HfO_2_ nanoparticle concentrations and for Au nanoparticles, a slightly increased cell viability was observed for the 0 Gy sham‐irradiation compared to the nanomaterial‐free control (Figure S10, Supporting Information). Previous reports on nanomaterial radioenhancement in NHDF cells using a 150 kVp X‐ray source have shown that neither MOFs and MXenes, nor metal oxides or Au nanoparticles led to observable radiosensitization.^[^ ^5^, ^20^, ^21^ ^]^ Reasons for the lower radiosensitization potential in NHDF cells compared to cancerous cells might be a higher antioxidant capacity of normal cells, resulting in lower sensitivity to increased production of ROS,^[^ ^72^ ^]^ and/or lower replication stress and DNA repair defects in normal cells compared to tumor cells.^[^ ^73^ ^]^ Although cancer cells generally exhibit higher basal levels of ROS, which activate antioxidant pathways and increase the ROS clearance rate,^[^ ^74^, ^75^ ^]^ they are more susceptible to therapies that further induce ROS, making them more sensitive to oxidative stress compared to their normal counterparts.^[^ ^72^, ^76^, ^77^, ^78^ ^]^ These results indicate that Ti‐based nanomaterials widen the therapeutic window for clinical radiotherapy.

Taken together, Ti‐based nanomaterials show promising potential to enhance the efficacy of clinical cancer radiotherapy. While biological effects of nanomaterials nowadays are still difficult to design and can depend on cell line and cellular environment, radiocatalytic properties of nanomaterials are inherent, can be assessed cell‐free and can be compared to each other to guide material design principles.

**Figure 9:**
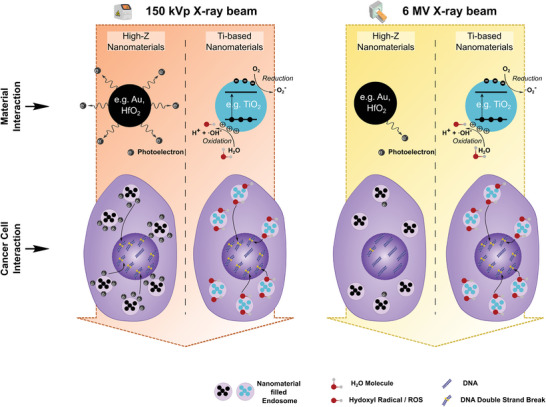
Schematic illustration of the mechanistic radioenhancement process of high‐Z versus Ti‐based nanomaterials for a pre‐clinical 150 kVp and clinical 6 MV X‐ray source. The “Material Interaction” highlights the Photoelectron or catalytic ROS generation during the material‐radiation interaction for high‐Z versus Ti‐based nanomaterials, respectively. Differences in Photoelectron generation are depicted for 150 kVp and 6 MV X‐ray interactions due to X‐ray energy dependent effects. The “Cancer Cell Interaction” highlights the Photoelectron or ROS generation from endosome‐contained nanomaterial agglomerates within the cell cytoplasm and shows increased or less increased states of DNA double strand breaks in the cell nucleus due to the cell‐radiation interaction.

Pure TiO_2_ nanoparticles are excellent photocatalysts with a strong oxidative degradation ability of organic compounds, due to the sufficiently positive valence band potential allowing for the production of **∙**OH radicals.^[^ ^9^ ^]^ In comparison to TiO_2_, similarly sized HfO_2_ nanoparticles showed less ROS production under X‐ray irradiation.^[^ ^31^ ^]^ For high‐Z nanoparticles such as HfO_2_, an influence of the X‐ray energy on ROS generation is expected, and is indicated by our data, due to increased Auger and photoelectron production at keV compared to MeV X‐ray energies (**Figure** **9**). This is because the photoelectric effect, whose cross‐section scales roughly with the atomic number Z and the incident photon energy E as σ_PE_ ≈Z^4^/E^3^, dominates at keV photon energies and preferentially creates inner‐shell electron vacancies that are typically filled via the Auger effect.^[^ ^79^ ^]^ The Pt‐decorated TiO_2_ nanoparticles in our study showed increased X‐ray induced ROS generation compared to pure TiO_2_ nanoparticles. This is further highlighted by the fact that their primary particle size was bigger (and, thus, their specific surface area was smaller) than that of the nondecorated counterpart, while catalytic ROS production scales with the inverse of the particle diameter.^[^ ^80^ ^]^ It has been shown that the Pt decoration of TiO_2_ nanoparticles enhanced the photocatalytic activity under ultraviolet light irradiation, possibly due to an enhanced charge separation.^[^ ^36^ ^]^ Also, the decoration of TiO_2_ nanoparticles with Ag has been shown to improve the photocatalytic performance, for example, in the degradation of methyl orange,^[^ ^81^ ^]^ or the oxidation of sucrose.^[^ ^82^ ^]^ However, in a direct comparison, it was shown that the photocatalytic hydrogen production performance of Pt‐decorated TiO_2_ nanoparticles was higher than that of Ag‐decorated TiO_2_ nanoparticles, in accordance with the formation of a higher Schottky barrier preventing charge recombination.^[^ ^38^ ^]^ Decreased ROS generation of TiO_2_:Ag compared to TiO_2_ under X‐ray generation, might, in our case, be due to the slightly bigger primary particle size, which leads to a lower accessible surface area. These results let us conclude that TiO_2_ consistently leads to high ROS production in a largely incident‐energy independent way, and that ROS production can be further increased by bandgap engineering, including noble metal decoration of the TiO_2_.

In these cell‐free tests, MIL‐125 MOFs were a most effective catalytic ROS generator, consistent with its previously documented strong photocatalytic activities under solar^[^ ^83^ ^]^ and UV irradiation.^[^ ^84^ ^]^ The photocatalytic mechanism of MIL‐125 is based on photoinduced charge separation,^[^ ^14^ ^]^ similar to semiconductors. Interestingly, PCN‐415 MOFs did not show comparable ROS generation, despite their known strong catalytic activity toward water reduction.^[^ ^85^ ^]^ Under kVp irradiation, both MIL‐125 and PCN‐415 demonstrated similar levels of ROS generation at the same mass concentration when in triethylammonium acetate buffer.^[^ ^20^ ^]^ A separate study indicated that PCN‐415 MOFs are not fully stable in PBS, which could explain their low ROS generation in our tests, potentially due to partial phosphate transformation similar to ZIF‐8 or MIL‐100(Fe) MOFs.^[^ ^86^, ^87^, ^88^ ^]^

TiN nanoparticles are conductive plasmonic nanoparticles, which possess a similar LSPR (localized surface plasmon resonance) performance to Au.^[^ ^89^ ^]^ This property, however, did not lead to ROS enhancement under X‐ray irradiation in our study. As expected, no ROS enhancement was found for SiO_2_ nanoparticles, which were used as negative control particles due to their very large bandgap and their amorphous structure hindering electron and hole mobility.^[^ ^90^ ^]^

## 3 Conclusions

Here, we present the radioenhancement performances of a selection of Ti‐containing nanomaterials under clinically relevant radiotherapy conditions. Experiments on Ti‐based nanomaterials reveal that radioenhancement effects of Ti‐containing materials are largely independent of the X‐ray energy, in stark contrast to those of high‐Z materials (Au and HfO_2_). These strong radioenhancement effects can be attributed to catalytic hydroxyl radical formation by the Ti‐based materials. In fact, Ti‐based materials displayed significantly better enhancement properties than Au or HfO_2_ nanoparticles at comparable particle doses and uptakes for clinically most widely used megavoltage X‐rays. Interestingly, emerging classes of Ti‐containing nanomaterials, such as MOFs and MXenes, showed especially promising effects in direct benchmarking experiments. Given that no radioenhancement effects were observed in normal noncancerous (NHDF) cells, even at a nanoparticle uptake comparable to that of the cancer cells, renders nanomaterial radioenhancement a highly attractive method for increasing the therapeutic window of radiotherapy. Such a therapy may, in the future, enable the reduction of the overall X‐ray dose needed to eradicate tumor tissue and, hence, make radiotherapy applicable to patients with radiosensitive as well as radioresistant tumors.

## 4 Experimental Section

### Materials

The reagents titanium(IV) isopropoxide (TTIP, Sigma‐Aldrich, 97%), 2‐ethylhexanoic acid (2EHA, Sigma‐Aldrich, ≥ 99%), platinum(II) acetylacetonate (Sigma‐Aldrich, 97%), xylene (VWR Chemicals, ≥96%), terephthalic acid (Sigma‐Aldrich, 98%), and trifluoroacetic acid (Sigma‐Aldrich, ≥ 99%) were used as received without further purification. Oxygen and methane gases were provided by PanGas (purity > 99%). The 20 nm TiN nanoparticles were supplied by PlasmaChem GmbH (PL‐HK‐TiN, Berlin, Germany) as a dry powder. Citrate stabilized 50 nm Au nanoparticles were supplied by nanoComposix Inc. (San Diego, CA) dispersed in USP purified water at 1 mg mL^−1^, with an endotoxin concentration of < 2.5 EU mL^−1^ (BioPure).

### Nanomaterial Synthesis

For the synthesis of inorganic nanoparticles via liquid‐feed flame spray pyrolysis (FSP), first the metal precursor solutions were prepared and then fed through a thin spray nozzle where the liquid precursor solution was dispersed into fine droplets by a 5 L min^−1^ oxygen gas flow. The pressure drop at the spray nozzle was 1.5 bar. The methane/oxygen feed ratio to sustain the spray flame was 1.5/3.2 L min^−1^. The setup is described elsewhere.^[^ ^91^ ^]^ For the synthesis of TiO_2_ nanoparticles, 10.23 g of TTIP were dissolved in 214.34 mL of xylene, reaching a total metal concentration of 0.16 m. The nanoparticle precursor solution was fed with a velocity of 3 mL min^−1^ through the spray nozzle.^[^ ^5^ ^]^ For the synthesis of Ag supported on TiO_2_ nanoparticles (TiO_2_:Ag), 3.39 g of TTIP and 24.90 mg of silver acetate were dissolved in 35.73 mL of 2EHA and 35.73 mL of acetonitrile, reaching a total metal concentration of 0.16 m and an Ag/Ti atom ratio of 1.25%.^[^ ^92^ ^]^ The nanoparticle precursor solution was fed with a velocity of 8 mL min^−1^ through the spray nozzle. For Pt supported on TiO_2_ nanoparticles (TiO_2_:Pt), 3.80 g of TTIP was dissolved in a mixture of 40 mL 2EHA and 40 mL acetonitrile, reaching a final metal concentration of 0.167 m. Then, 0.24 g of Pt acetylacetonate was added, reaching a final Pt/Ti atom ratio of 4.5%. The nanoparticle precursor solution was fed with a velocity of 8 mL min^−1^ through the spray nozzle.^[^ ^37^ ^]^ After FSP synthesis, nanoparticles were collected on a glass fiber filter and sieved (mesh size 0.25 mm) before use.

Metal organic frameworks Ti‐MIL‐125 and Ti/Zr‐PCN‐415 were synthesized as reported elsewhere and collected as a dry powder using a vacuum oven.^[^ ^85^, ^93^ ^]^ In short, for the synthesis of Ti‐MIL‐125, 1.375 g of 2‐aminoterephtalic acid (Sigma‐Aldrich) was stirred for 40 min in 20 mL of DMF and 5 mL of methanol. The solution was then heated in an oil bath at 110 °C for 10 min. To this, 1.5 mL of TTIP and 0.1 mL of Milli‐Q were added under continuous vigorous stirring and the solution was left to react for 32 h. The Ti‐MIL‐125 MOF precipitate was washed once with DMF and then twice with ethanol using centrifugation (11 000 g, 4 °C, 45 min). For the synthesis of Ti/Zr‐PCN‐415, 50 mg of ZrCl_4_ were dissolved by stirring in 5 mL of DMF. Then, 0.5 mL of acetic acid and 0.1 mL of TTIP were added under continued stirring. After 10 min, the reaction flask was sealed and heated in an oven (100 °C, 24 h) without further stirring. A mixture of 800 mg of terephtalic acid in 10 mL of DMF was added to the reaction flask under stirring. Thereafter, 1 mL of trifluoroacetic acid was added and the mixture was stirred for another 5 min. After transferring the mixture into a Teflon lined autoclave, it was heated for 24 h at 140 °C. After cooling, the white precipitate was washed once with DMF and then twice with ethanol using centrifugation (11 000 g, 4 °C, 45 min). Both MOF precipitates were finally dried in a vacuum oven at 40 °C.

The synthesis of a colloidal Ti_3_C_2_T*_x_* MXene suspension was performed as reported in literature.^[^ ^94^, ^95^ ^]^ In short, 1 g of Ti_3_AlC_2_ powder (Carbon‐Ukraine) was added to a mixture of 6 mL of DI water, 12 mL of HCl and 2 mL of HF. After stirring the mixture for 24 h (25 °C, 300 rpm), the multilayer Ti_3_C_2_T*_x_* powder was washed with DI water repeatedly via centrifugation (3500 rpm, 5 min) until a neutral pH was reached. The Ti_3_C_2_T*_x_* multilayers were delaminated by stirring in 1 g of lithium chloride (LiCl, Carl Roth, 99%) and 50 mL of DI water for 24 h (300 rpm, 25 °C). Thereafter, the solution was centrifuged (3500 rpm, 10 min) repeatedly until the supernatant became dark. MXene flake solutions were stored in a freezer at −20 °C to reduce the amount of sample oxidation. While we did not observe the presence of titania in the XRD pattern for MXenes stored at −20 °C, TEM analysis showed the sparse presence of small titania nanoparticles on the MXene sheets, indicating a low degree of surface oxidation (Figure 1a). This is in agreement with prior reports showing that MXene sheets stored in aqueous dispersions are slowly oxidized, which results in the formation of titania.^[^ ^96^, ^97^, ^98^ ^]^ A high degree of oxidation, which was accompanied by a visual color change of the MXene solution from black to gray‐white, was only observed when the MXene dispersion was stored at room temperature (or at even higher temperatures) for several days. In this case, XRD Rietveld analysis (Table S1, Supporting Information) confirmed the presence of 15.1 ± 0.2 nm sized rutile nanoparticles. Thus, only fresh MXenes stored at −20 °C were used for the following experiments.

### Nanoparticle Dispersion Preparation

All nanoparticle solutions were freshly prepared before the start of each experiment and sonicated for ≈30 min. Dry nanoparticle powders were suspended in Milli‐Q water.

### Nanoparticle Characterization

For crystal phase and grain size analysis, XRD patterns were collected from the sample powder using a Bruker D8 Discovery or a Bruker D2 Phaser diffractometer, and Rietveld refinement was undertaken using Profex^[^ ^99^ ^]^ (Version 4.3.5). Only in the case of Ti_3_C_2_ MXene and Au nanomaterials, which were stored as solutions, liquids were dropped on the XRD sample holder and dried in a vacuum oven (30 °C) before collecting the XRD pattern. To assess the morphology of the nanoparticles, transmission electron microscopy (TEM) imaging was performed using a JEOL 2200FS electron microscope operated at 200 kV or a Zeiss EM 900 electron microscope operated at 80 kV. High resolution STEM imaging was performed on a Hitachi HD‐2700 Cs‐STEM. In the case of MIL‐125 and PCN‐415, where the XRD grain size was not accessible, the diameter of the MOFs was measured individually from TEM images using ImageJ (U.S. National Institutes of Health, Bethesda, MD). The hydrodynamic diameter (z‐Average) and Zeta‐Potential was determined by DLS measurements using a Zetasizer Nano ZS90 instrument (Malvern Instruments Ltd., Worcestershire, UK) with a 90° scattering angle. Size measurements in different media (MilliQ water, PBS, phenolred‐free MEM + 10%FCS) were performed by diluting and mixing bath‐sonicated, water‐based nanomaterial stock solutions 10x in the respective medium. Zeta‐Potential measurements were performed by diluting nanomaterial stock solutions 10x in MilliQ with additional 2% PBS, leading to a conductivity of 0.3–0.4 mS cm^−1^.

### Irradiation Set‐Up

To irradiate cells or nanomaterial solutions, the same irradiation conditions as those in ref. [31] were used. Cell plates, or Eppendorf tubes sitting in a PMMA inset, were placed within the central recess of a PMMA phantom (8 × 40 × 40 cm^3^) consisting of two slabs of equal size. A kVp tube source (Seifert ISOVOLT 450, GE Sensing & Inspection Technologies GmbH, Germany) with a 7 mm beryllium filter window was positioned 50 cm above the bottom phantom slab and operated at 150 kV and 20 mA to give a dose rate of 1.5 Gy min^−1^ at the location of the central recess. A calibrated ionization chamber (N31003, PTW, Freiburg, Germany) connected to a UNIDOS dosimeter (PTW, Freiburg, Germany) ensured the dose supply during irradiation. For MV X‐ray irradiation at the hospital, a clinical linear accelerator (TrueBeam, Varian, Paolo Alto, CA) operating at 6 MV using a flattening filter supplied photons to the samples with a dose rate of 5.7 Gy min^−1^ at a field size of 20 × 20 cm^2^.

### ROS Assay

To measure ROS generation in nanomaterial solutions, the DCF assay was adapted from the protocols of Zhao and Riediker.^[^ ^100^ ^]^ 2′,7′‐Dichlorodihydrofluorecein diacetate powder (H_2_DCF‐DA, Sigma‐Aldrich) was dissolved in DMSO (dimethyl sulfoxide, Sigma‐Aldrich) at a concentration of 5 mm, and 12 µL aliquots of this were stored at −20 °C, protected from light, for further use. To activate the H_2_DCF‐DA stock, it was incubated with 48 µL of fresh NaOH (10 mm) for 30 min in the dark at room temperature. Thereafter, it was diluted 1:100 with PBS buffer to reach a 10 µm fluorophore concentration. This working solution was kept in the dark at all times. All nanomaterials were weighted and sonicated in Milli‐Q water at 2 mg mL^−1^. Prior to irradiation with 0 or 12 Gy, 0.1 mL of H_2_DCF working solution was added to 0.1 mL of 2, 1, 0.5, or 0 mg mL^−1^ nanomaterial‐suspension. Following irradiation, all tubes were centrifuged at 21 000 g for 10 min at 14 °C and 40 µL triplicates of the supernatant were transferred to a transparent 96‐well plate, which was kept in the dark and cooled until the collection of the fluorescence signal using a microplate reader (485/535 nm excitation/emission, Mithras LB 943 Multimode). The chemical dose‐enhancement factors were calculated by dividing the change in fluorescence intensity (FI) after irradiation (Δ = FI_12Gy_‐FI_0Gy_) of nanomaterial solutions by that of nanomaterial‐free solutions.

### Cell Lines and Cell Handling

The human fibrosarcoma cell line HT1080 (ATCCCCL121TM) was cultured in minimum essential medium Eagle (MEM, Sigma‐Aldrich or Gibco) supplemented with 10% fetal calf serum (FCS, Sigma‐Aldrich), 1% L‐glutamine (L‐glut, Sigma‐Aldrich), 1% penicillin‐streptomycin (PS, Sigma‐Aldrich), 1% nonessential amino acids (NEAA, Sigma‐Aldrich), and 1 mm sodium pyruvate. The normal human dermal fibroblast cell line NHDF (PromoCell C‐12302) was cultured in Dulbecco's modified Eagle's medium (high glucose, Sigma‐Aldrich) supplemented with 10% FCS, 1% L‐glutamine, and 1% penicillin‐streptomycin. All cells were kept at 37 °C under a humidified, 5% CO_2_ containing atmosphere and were subcultured at 70%–80% confluency by treatment with 0.5% Trypsin‐EDTA (Sigma‐Aldrich).

### Cell Viability Assay

To assess the nanoparticle cell compatibility, 5000 HT1080 or NHDF cells were seeded in black 96 well plates with a transparent bottom in 100 µL of growth medium and were allowed to attach for 24 h. Nanomaterial treatment was performed in triplicates by replacement of the cell medium with 100 µL of nanoparticle added medium suspensions in increasing concentrations of 0, 10, 20, 50, 100, 200, and 500 µg mL^−1^. The total water content of all nanoparticle‐medium suspensions was 10%. After 24 h of nanoparticle treatment, the cell viability assay was performed according to the manufacturer protocol of the CellTiter‐Glo (CTG) Luminescent Cell Viability Assay (Promega, Switzerland). The nanoparticle‐medium suspension was aspirated and replaced by 50 µL of fresh cell medium and 50 µL of CellTiter‐Glo reagent. After 10 min of shaking in the dark, luminescence was measured using a Mithras LB 943 Multimode plate reader.

### In Vitro Irradiation

To investigate the radioenhancing nanomaterial properties in vitro, HT1080 or NHDF cells were seeded at a density of 2000 cells in 300 µL of full growth medium in 48‐well plates. On the following day, 200 µL of nanomaterials in the full growth medium were added to the cells, reaching final nanomaterial concentrations of 5, 10, 20, 50, 100, 200, or 300 µg mL^−1^. Nanomaterials were previously weighted into Milli‐Q water and sonicated for 30 min, and the final water content in each well was 10%. After 24 h of nanomaterial incubation, cells were washed twice with 250 µL of PBS before 500 µL of the full growth medium was added to each well. Thereafter, cells were transported to the irradiation facilities for roughly 1 h in a cooled styrofoam box. Cells were treated with 0, 3, or 6 Gy at room temperature. After being transported from the irradiation facilities, cells were kept in a 37 °C incubator. The cell medium was replaced after 3 days and a cell viability assay (CTG) was performed 5 (HT1080, doubling time ≈ 18 h) or 7 (NHDF, doubling time ≈32 h) days after irradiation by replacing 280 µL of the cell medium with 200 µL of CTG reagent, 20 min of shaking and 10 min of equilibration in the dark. Luminescence was measured with a Mithras LB 943 Multimode plate reader.

For the HT1080 cell surviving fraction analysis after X‐ray treatment, the luminescence signal was translated into the number of cells using a sigmoidal fit from a cell standard curve via

(2) $$\#\mathrm{cells}\;=\frac{y\;b}{a-y}\;$$

where *y* is the luminescence value, and *a* and *b* are the fitting parameters from the luminescence standard curve (Figure S11, Supporting Information). The cell standard curve was performed using a similar cell growth medium (≈250 µL) to CTG reagent (200 µL) ratio as compared with a cell irradiation experiment and was performed prior to the analysis of irradiation experiments. For NHDF cells, a linear luminescence to cell number relationship was assumed, since the relative luminescence values were < 1 × 10^6^.

The cell surviving fraction (SF) after X‐ray treatment was calculated by normalizing the cell number after irradiation either to the nanomaterial‐free control cells without irradiation (which included nanomaterial (NM) effects without irradiation)

(3) $$\mathrm{SF}\left(x\;\mathrm{Gy}\right)=\frac{{\left(\#\mathrm{cells}\right)}_{\mathrm{with}\;\mathrm{or}\;\mathrm{without}\;\mathrm{NMs}\;\mathrm{at}\;x\;\mathrm{Gy}}\;}{{\left(\#\mathrm{cells}\right)}_{\mathrm{without}\;\mathrm{NMs}\;\mathrm{and}\;\mathrm{without}\;X-\mathrm{ray}}}\;$$

or by normalizing the cell number after irradiation to the same condition without irradiation

(4) $$\mathrm{SF}\left(x\;\mathrm{Gy}\right)=\frac{{\left(\#\mathrm{cells}\right)}_{\;\mathrm{with}\;\mathrm{or}\;\mathrm{without}\;\mathrm{NMs}\;\mathrm{at}\;x\;\mathrm{Gy}}}{{\left(\#\mathrm{cells}\right)}_{\mathrm{with}\;\mathrm{or}\;\mathrm{without}\;\mathrm{NMs}\;\mathrm{and}\;\mathrm{without}\;X-\mathrm{ray}}}\;$$

To the surviving fraction, a linear quadratic fit was performed via

(5) $$\mathrm{SF}\left(x\;\mathrm{Gy}\right)=C\;e^{-\alpha x-\beta x^{2}}\;$$

where α, β, and C are fitting parameters. The in vitro radioenhancing effect (dose modifying ratio, DMR) of the nanomaterials was calculated by dividing the X‐ray doses that lead to the same effect of 50% cell survival (LD_50_) without nanomaterials by the ones with nanomaterials.

(6) $$\mathrm{DM}R_{50\%}=\frac{LD_{\;50\;\left(\mathrm{without}\;\mathrm{NM}\right)}}{LD_{50\;\left(\mathrm{with}\;\mathrm{NM}\right)}}\;$$

### γH2AX Staining, Immunofluorescence, and Quantification

Cells were grown on cover slips in 24‐well plates. 30 min or 24 h after X‐ray irradiation, cells were washed twice with PBS before fixation with 3% PFA for 15 min at room temperature (RT). Next, cells were washed twice for 5 min and permeabilized with 2% BSA and 0.3% Triton X‐100 in PBS for 1h at RT. After washing twice, cells were incubated overnight at 4 °C with primary rabbit antiphospho‐Histone H2AX antibody (Cell Signaling Technology, #9718, 1:750) in 1% BSA and 0.3% Triton X‐100 in PBS. Subsequently, cells were washed three times for 10 min with PBS and incubated with secondary goat antirabbit Alexa Fluor 488 secondary antibody (Invitrogen, #A11070, 1:1200) and Hoechst 33 342 (MedChemExpress, HY‐15559A, 5 µg mL^−1^) for at least 1 h in 1% BSA and 0.3% Triton X‐100 at RT in the dark. After washing three times, coverslips were mounted on microscope glass slides and images were acquired using a Zeiss Elyra 7 microscope with lattice SIM (structured illumination microscopy) super‐resolution module. For each field, z‐stacks were combined in one representative image for quantification and image representation using maximum intensity projection (MIP). For both lattice SIM image processing and MIP, ZEN black edition software was used. γH2AX foci counting and mean intensity measurements were performed with CellProfiler 4.0 by applying a custom‐adapted speckle counting pipeline in which Otsu thresholding was used for foci recognition (pipeline available as Supporting Information). Cells with pan‐nuclear γH2AX staining were excluded from the analysis.

### Electron Microscopy Imaging and Metal Uptake Quantification of Nanomaterials in Cells

To investigate the nanomaterial uptake into cells, 150 000 HT1080 or 450 000 NHDF cells were seeded in a T25 flask and left to attach for 24 h. Thereafter, cells were treated with a 0.1 mg mL^−1^ (in the case of metal oxides/nitride or Au nanoparticles), 0.05 mg mL^−1^ (in the case of MOFs), or 0.02 mg mL^−1^ (in the case of Ti_3_C_2_T*_x_* MXenes flakes) fresh nanoparticle/cell‐medium solution containing 10% H_2_O. After a 24 h nanoparticle treatment, cells were washed with PBS and detached from the flasks using 0.5–0.8 mL Trypsin‐EDTA. After stopping the trypsin reaction using 1.2–1.5 mL of cell medium, trypsin was removed by centrifugation (5 min, 200 g) and the cells were re‐suspended in a total of 1 mL of fresh cell medium.

For metal uptake quantification, cells were then counted and 100 000–400 000 cells were stored as cell pellets in the freezer (−20 °C) until digestion, after removing the cell medium by centrifugation (5 min, 200 g). All cell samples containing Au nanoparticles were digested at room temperature by suspending them in 4 mL of aqua regia and 0.1 mL of H_2_O_2_ for 2.5 h. All other materials were digested at room temperature by incubating the sample in 2 mL of HNO_3_, 1 mL of H_2_O_2_, and 0.3 mL of HF for 2 h before diluting it with Milli‐Q water. For ICP‐OES analysis with an Agilent 5110 ICP‐OES instrument (Agilent, Switzerland), samples contained 2% HNO_3_. Samples containing Au were analyzed in additional 1% L‐cysteine in order to coordinate the Au atoms and decrease any carryover during analysis. The metal standards for calibration were prepared in the same matrix as the samples.

For imaging cells with electron microscopy, cells were fixed by replacing the cell medium with 2.5% glutaraldehyde (Sigma‐Aldrich) in 0.1 m sodium cacodylate buffer (Electron Microscopy Sciences). After 1 h of fixation at room temperature, cell pellets were washed twice with 0.1 m sodium cacodylate buffer for 3 min. Thereafter, cell pellets were stained with 1% osmium tetroxide (Electron Microscopy Sciences) in 0.1 m sodium cacodylate buffer for 1 h at room temperature. Subsequently, 5 min of ethanol dehydration washing steps (30%, 50%, 70%, and 90% ethanol) were performed, followed by three 10‐min 100% ethanol incubations. Finally, the cell pellets were left for 1 h in a 1:1 ethanol to Epon (Epoxy embedding kit 45 359, Sigma‐Aldrich, Germany) mixture, and then overnight in 100% Epon before they were polymerized in fresh Epon at 60 °C for 48 h. Ultrathin sections of 60–80 nm thickness were cut with an ultramicrotome (Leica EM UC6, Germany) and TEM images were recorded with a Zeiss EM 900 transmission electron microscope (Carl Zeiss Microscopy GmbH, Germany) at 80 kV.

To investigate cellular nanomaterial uptake in irradiated cells, cells were treated like in an irradiation experiment. After nanomaterial washing, prior to irradiation, cells were harvested by 10 min trypsination (with 80 µL Trypsin‐EDTA). Triplicates were then transferred using 220 µL fully supplemented growth medium per well and pooled into one Eppendorf tube. Cells were spun down and transferred into a smaller 200 µL tube. After washing the cell pellet, cell samples were transferred into 50 mL Falcon tubes in a total of 80 µL cell medium. Four control samples were counted using a hemocytometer. Each cell sample was then digested using aqua regia or HF and analyzed using ICP‐OES as stated above.

## Conflict of Interest

The authors declare no conflict of interest.

## Author Contributions

L.R.H.G. contributed to the study design, performed experiments, analyzed data, and drafted the manuscript. C.B. performed radiobiological experiments and imaging. B.A.B. helped with radiobiological experiment protocols. V.M.K. performed sample preparation for TEM including embedding, ultrathin sectioning, and grid making, as well as TEM imaging and data compilation. A.G. developed and performed elemental analysis protocols. S.W. and M.R.L. synthesized and characterized MXenes. H.S. helped with irradiation experiments and verified the dose supply during photon irradiation. M.P. supervised the radiobiological experiments. L.P. provided input at the clinical irradiation facilities. I.K.H. conceived and supervised the study and edited the manuscript. All authors contributed to the manuscript writing and have given approval to the final version of the manuscript.

## Supporting information



Supporting Information

Supporting Information

## Data Availability

The data that support the findings of this study are available from the corresponding author upon reasonable request.
