# Supplementary material for: Catalytically Active Ti‐Based Nanomaterials for Hydroxyl Radical Mediated Clinical X‐Ray Enhancement
Source: Adv Sci (Weinh). 2024 Nov 5;11(47):2406198. doi: 10.1002/advs.202406198 (PMC11653640; doi:10.1002/advs.202406198)
Supplement: Supplementary file 1 — Supporting Information [file ADVS-11-2406198-s001.pdf]

ADVANCED SCIENCE
Open Access

Supporting Information

for *Adv. Sci.*, DOI 10.1002/advs.202406198

Catalytically Active Ti-Based Nanomaterials for Hydroxyl Radical Mediated Clinical X-Ray Enhancement

*Lukas R. H. Gerken, Claire Beckers, Beatrice A. Brugger, Vera M. Kissling, Alexander Gogos, Shianlin Wee, Maria R. Lukatskaya, Hans Schiefer, Ludwig Plasswilm, Martin Pruschy and Inge K. Herrmann**

SUPPLEMENTARY INFORMATION

Catalytically active Ti-based nanomaterials for hydroxyl radical mediated clinical X-ray enhancement

Lukas R.H. Gerken,[1,2] Claire Beckers,[3] Beatrice A. Brugger,[2] Vera M. Kissling,[2] Alexander Gogos,[1,2] Shianlin Wee,[4] Maria R. Lukatskaya,[4] Hans Schiefer,[5] Ludwig Plasswilm,[5,6] Martin Pruschy[3] and Inge K. Herrmann[1,2,7,8,]*

[1] Nanoparticle Systems Engineering Laboratory, Institute of Energy and Process Engineering (IEPE), Department of Mechanical and Process Engineering (D-MAVT), ETH Zurich, Sonneggstrasse 3, 8092 Zurich, Switzerland.

[2] Particles-Biology Interactions Laboratory, Department of Materials Meet Life, Swiss Federal Laboratories for Materials Science and Technology (Empa), Lerchenfeldstrasse 5, 9014 St. Gallen, Switzerland.

[3] Laboratory for Applied Radiobiology, Department of Radiation Oncology, University Hospital Zurich, University of Zurich, Winterthurerstrasse 190, 8057 Zurich, Switzerland.

[4] Electrochemical Energy Systems Laboratory, Institute of Energy and Process Engineering (IEPE), Department of Mechanical and Process Engineering (D-MAVT), ETH Zurich, Sonneggstrasse 3, 8092 Zurich, Switzerland.

[5] Department of Radiation Oncology, Cantonal Hospital St. Gallen (KSSG), Rorschacherstrasse 95, CH-9007 St. Gallen, Switzerland.

[6] Department of Radiation Oncology, Inselspital University Hospital, 3010 Bern, Switzerland

[7] The Ingenuity Lab, Balgrist University Hospital, Forchstrasse 340, 8008 Zurich, Switzerland.

[8] Faculty of Medicine, University of Zurich, Rämistrasse 71, 8006 Zurich, Switzerland.

*inge.herrmann@empa.ch; ingeh@ethz.ch; +41 (0)58 765 7153

Table S1: Rietveld refinement (grain size and phase refinement) of XRD patterns from FSP synthesized nanoparticles and the oxidized Ti_3C_2 MXene sheets. Data, when possible, given as refined value ± estimated SD (ESD).

**Nanoparticle**	**Refined Parameters**	**Phase 1**	**Phase 2**	**Phase 3**	**Refinement Statistics**	
TiO_2		***Anatase***	***Rutile***		-	
	**d_{XRD} (nm)**	6.7 ± 0.1	3.2		X^2	1.67
	**Phase (wt%)**	71 ± 1%	29 ± 1%		GOF	1.29
TiO_2:Ag		***Anatase***	***Rutile***	***FCC (Ag)***	-	
	**d_{XRD} (nm)**	7.5 ± 0.1	4.3 ± 0.2	1.6 ± 0.1	X^2	1.44
	**Phase (wt%)**	64 ± 1%	26 ± 1%	10 ± 1%	GOF	1.2
TiO_2:Pt		***Anatase***	***Rutile***	***FCC (Pt)***	-	
	**d_{XRD} (nm)**	8.5 ± 0.1	4.2	1.5 ± 0.2	X^2	0.18
	**Phase (wt%)**	60%	34%	6%	GOF	0.42
TiN		***cubic***			-	
	**d_{XRD} (nm)**	21.3 ± 0.2			X^2	1.51
	**Phase (wt%)**	100%			GOF	1.23
HfO_2		***Monoclinic***	***Orthorhombic***		-	
	**d_{XRD} (nm)**	5.6 ± 0.1	5.4 ± 0.5		X^2	1.88
	**Phase (wt%)**	77 ± 2 %	23 ± 2 %		GOF	1.32
TiO_2 in oxidized Ti_3C_2 MXene		***Rutile***				
	**d_{XRD} (nm)**	15.1 ± 0.2			X^2	1.16
	**Phase (wt%)**	100 %			GOF	1.08

Table S2: Zeta-potential and hydrodynamic sized of all nanomaterials dispersed in H_2O, PBS or 10% FCS supplemented, phenol red-free cell medium measured using DLS technique.

**Sample Name**	**Zeta-Potential** *(mV)*	**z-Average** *(nm)* **H_2O**	**PBS**	**Supplemented Medium**
TiO_2	-34.0 ± 0.4	137 ± 6	1460 ± 249	211 ± 4
TiO_2:Ag	-34.0 ± 0.3	266 ± 7	1037 ± 291	167 ± 9
TiO_2:Pt	-37.8 ± 0.4	1330 ± 16	188 ± 19	1645 ± 308
TiN	-28.2 ± 0.7	221 ± 3	1166 ± 193	187 ± 5
$Ti_3C_2T_x$ Mxene	-44.7 ± 0.6	1435 ± 197	1160 ± 328	491 ± 183
MIL-125	-27.4 ± 1.9	1102 ± 42	504 ± 4	436 ± 26
PCN-415	-51.0 ± 1.4	816 ± 11	382 ± 12	434 ± 10
HfO_2	-31.9 ± 1.6	143 ± 12	113 ± 1	168 ± 4
Au	-45.3 ± 1.6	53 ± 1	245 ± 42	67 ± 2

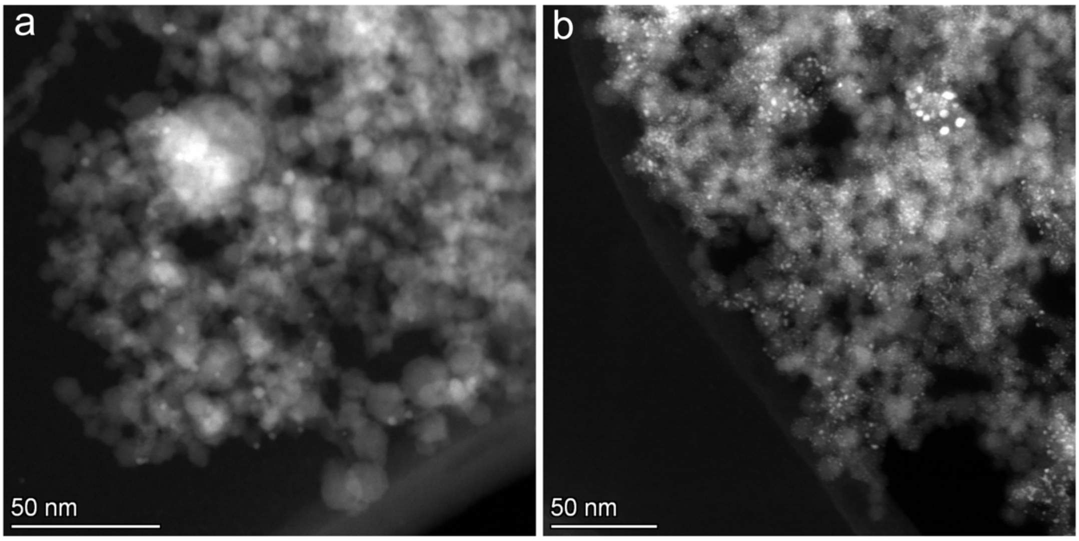

Figure S1: High Resolution STEM micrograph of Ag (a, TiO_2:Ag) and Pt decorated TiO_2 nanoparticles (b, TiO_2:Pt).

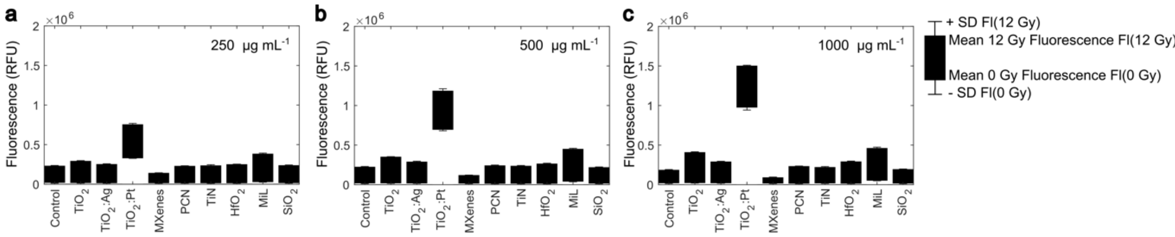

Figure S2: ROS generation during 12 Gy 150 kVp X-ray irradiation. Fluorescence measured by the H2DCF-DA assay for 250 (a), 500 (b) and 1000 µg/mL (c) nanoparticle solutions in 50% PBS buffer. Floating bar plots showing 0 Gy fluorescence (lower end of bar) and 12 Gy fluorescence values (Mean ± SD; n = 3). The amount or fluorescence (equivalent to the amount of ROS generated) can be enhanced by certain nanoparticles. Au nanoparticles could not be measured, due to severe background interaction of nanoparticle with fluorophore. (0 Gy fluorescence signal with Au NPs > $3.5x10^6$).

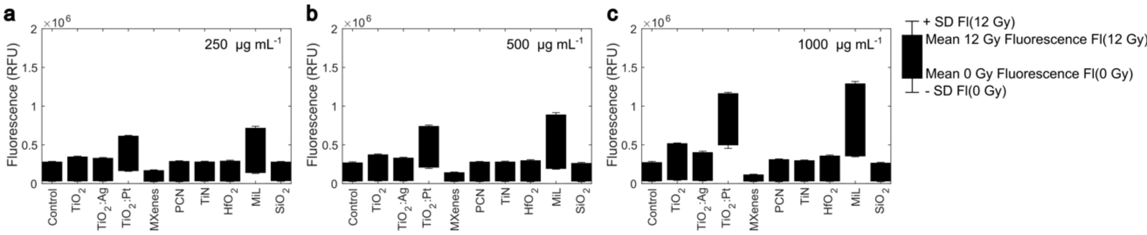

Figure S3: Reactive oxygen species (ROS) generation during 12 Gy 6 MV X-ray irradiation. Fluorescence measured by the H2DCF-DA assay for 250 (a), 500 (b) and 1000 µg/mL (c) nanoparticle solutions in 50% PBS buffer. Floating bar plots showing 0 Gy fluorescence (lower end of bar) and 12 Gy fluorescence values (Mean ± SD; n = 3). The amount or fluorescence (equivalent to the amount of ROS generated) can be enhanced by certain nanoparticles. Au nanoparticles could not be measured, due to severe background interaction of nanoparticle with fluorophore.

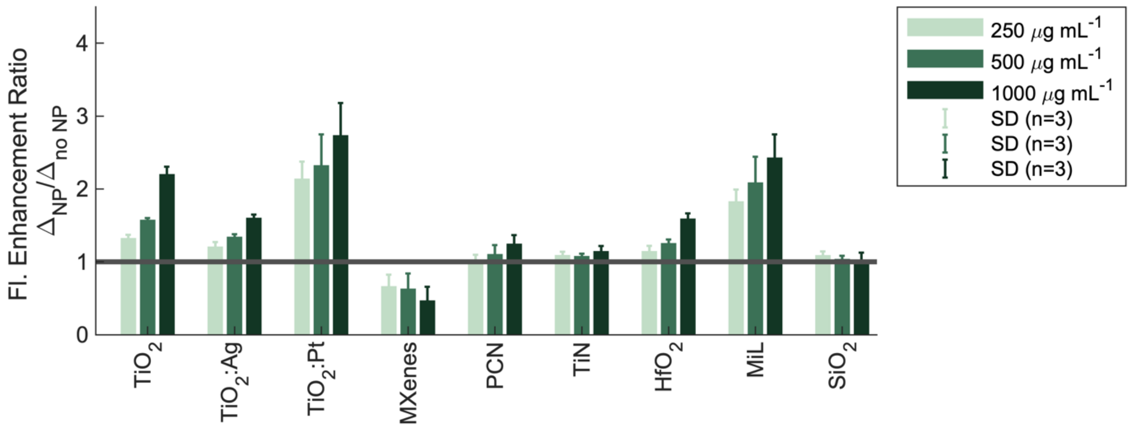

Figure S4: Acellular ROS enhancement quantification using the H_2DCF-DA assay. Nanoparticle (NP) ROS enhancement (displayed as a change in fluorescence before and after irradiation of nanoparticle fluorophore solutions compared to the fluorescence change for nanoparticle-free fluorophore solutions) after 12 Gy 150 kVp X-ray irradiation. Nanoparticles were tested at three different mass concentrations. Bars and error bars indicate the mean ± SD of the means of N = 4 independent experiments. Δ: change in fluorescence before and after 12 Gy X-ray irradiation of a 50% PBS solution with the H_2DCF fluorophore (with or without nanoparticles).

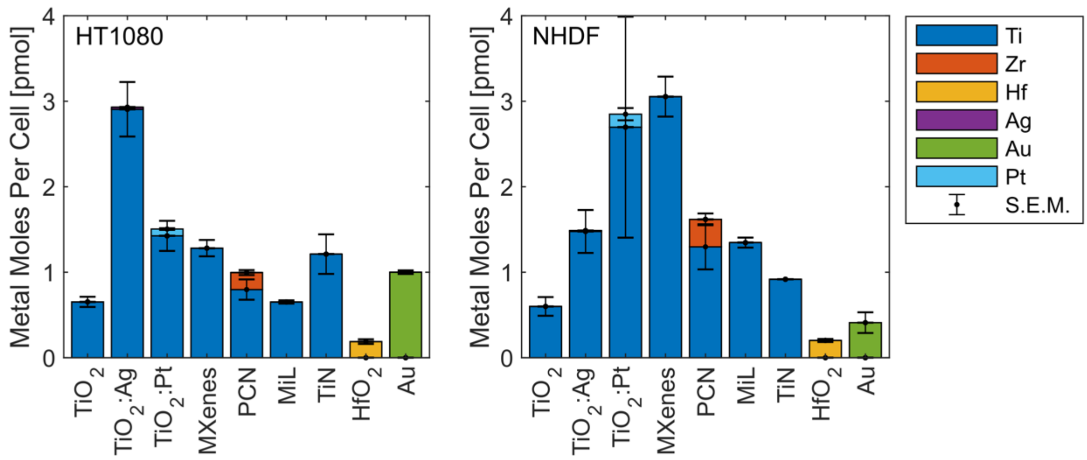

Figure S5: ICP-OES uptake quantification (in metal moles per cell) for all nanomaterials in HT1080 cancer cells and non-cancerous NHDF cells, with the same conditions as compared to TEM embedded samples. Before harvesting for analysis, cells were incubated for 24 hours with either 100 µg/mL (all metal oxides, TiN and Au nanoparticles), 50 µg/mL (MIL-125 or PCN-415 MOFs) or 20 µg/mL (Ti_3C_2 MXenes) of nanomaterial containing cell medium solutions.

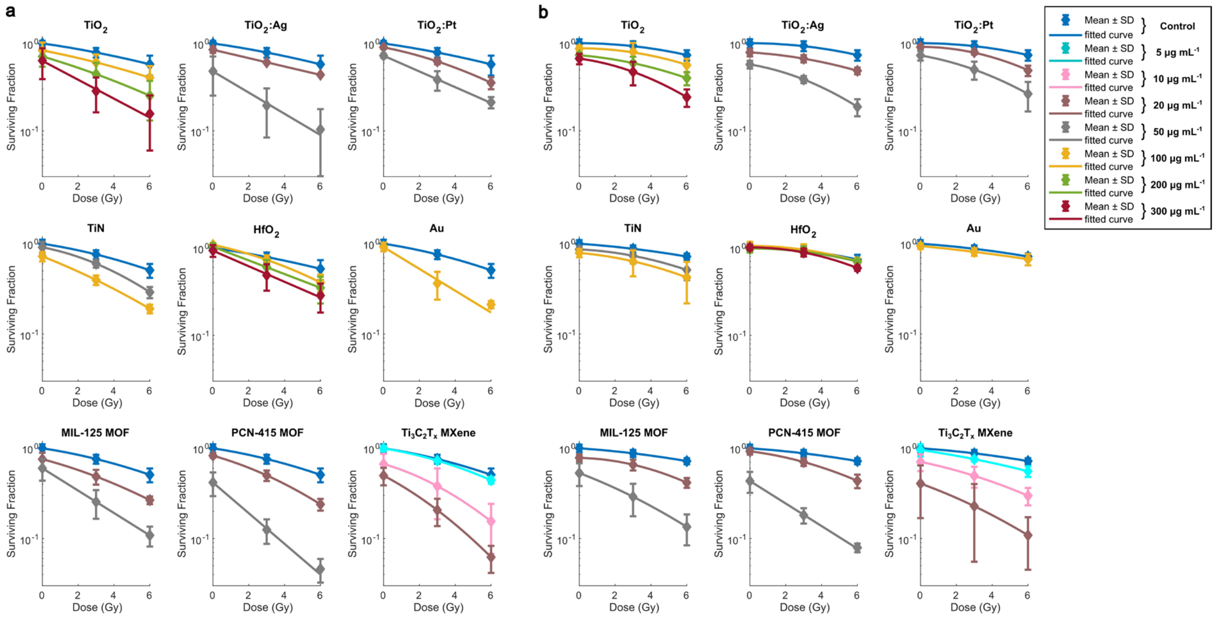

Figure S6: Survival curves (linear quadratic fit) of HT1080 cells incubated with different nanoparticles and concentrations and irradiated with 0, 3 and 6 Gy of 150 kVp (a) or 6 MV (b) X-rays. The surviving fractions were based on the metabolic activity of the cells 5 days post irradiation. All data points (mean ± SD, n = 6) were averaged from N = 2 independent biological experiments and were normalized to the nanomaterial-free control cell viability without irradiation.

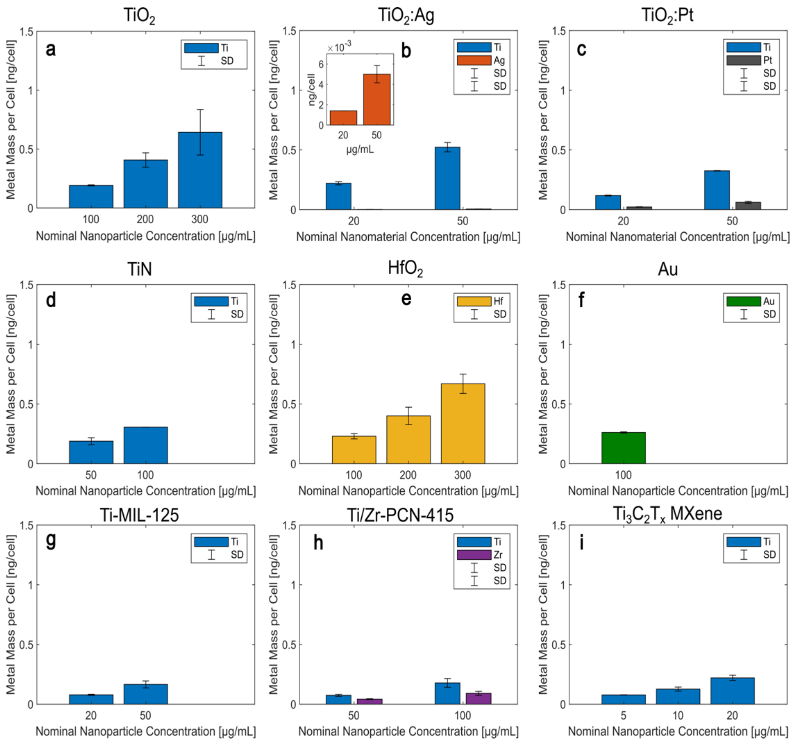

Figure S7: Metal mass uptake in HT1080 cells normalized to cell number (6000 cells per sample) as quantified by ICP-OES. Cells were treated with different concentrations (x-axis) of TiO_2 (a), Ag or Pt decorated TiO_2 (b and c), TiN (d), HfO_2 (e) or Au (f) nanoparticles, or with Ti-MIL-125 (g), Ti/Zr-PCN-415 (h) MOFs, or with $Ti_3C_2T_x$ MXenes (i) for 24 h. Cell samples were harvested for ICP-OES digestion after washing and before irradiating them with X-rays.

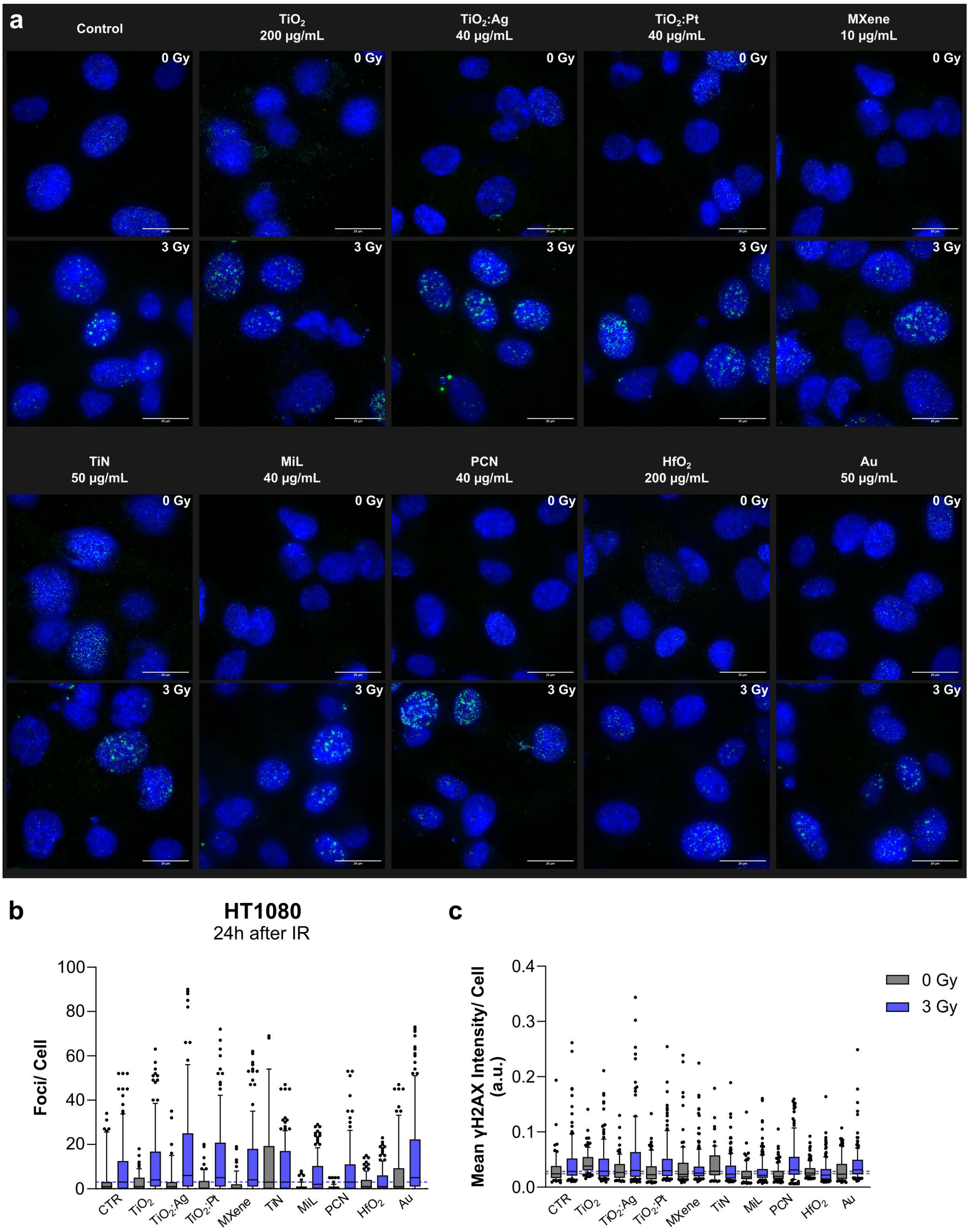

Figure S8: Representative immunofluorescence images showing γH2AX foci (green) in nuclei (blue) of HT1080 cells with and without nanomaterials 24 h after 0 or 3 Gy irradiation with 150 kVp X-rays (a); quantification of foci per nucleus (b) and mean γH2AX intensity per nucleus (c) for 0 and 3 Gy irradiated cells with and without nanomaterials; the whiskers indicate 10th and 90th percentiles; data points below and above the whiskers are drawn as individual points; boxes extend from the 25th to 75th percentiles; number of analyzed nuclei was around 80 per 0 Gy and around 110 per 3 Gy condition; increases in DNA damage compared to control (CTR) cells were not significant.

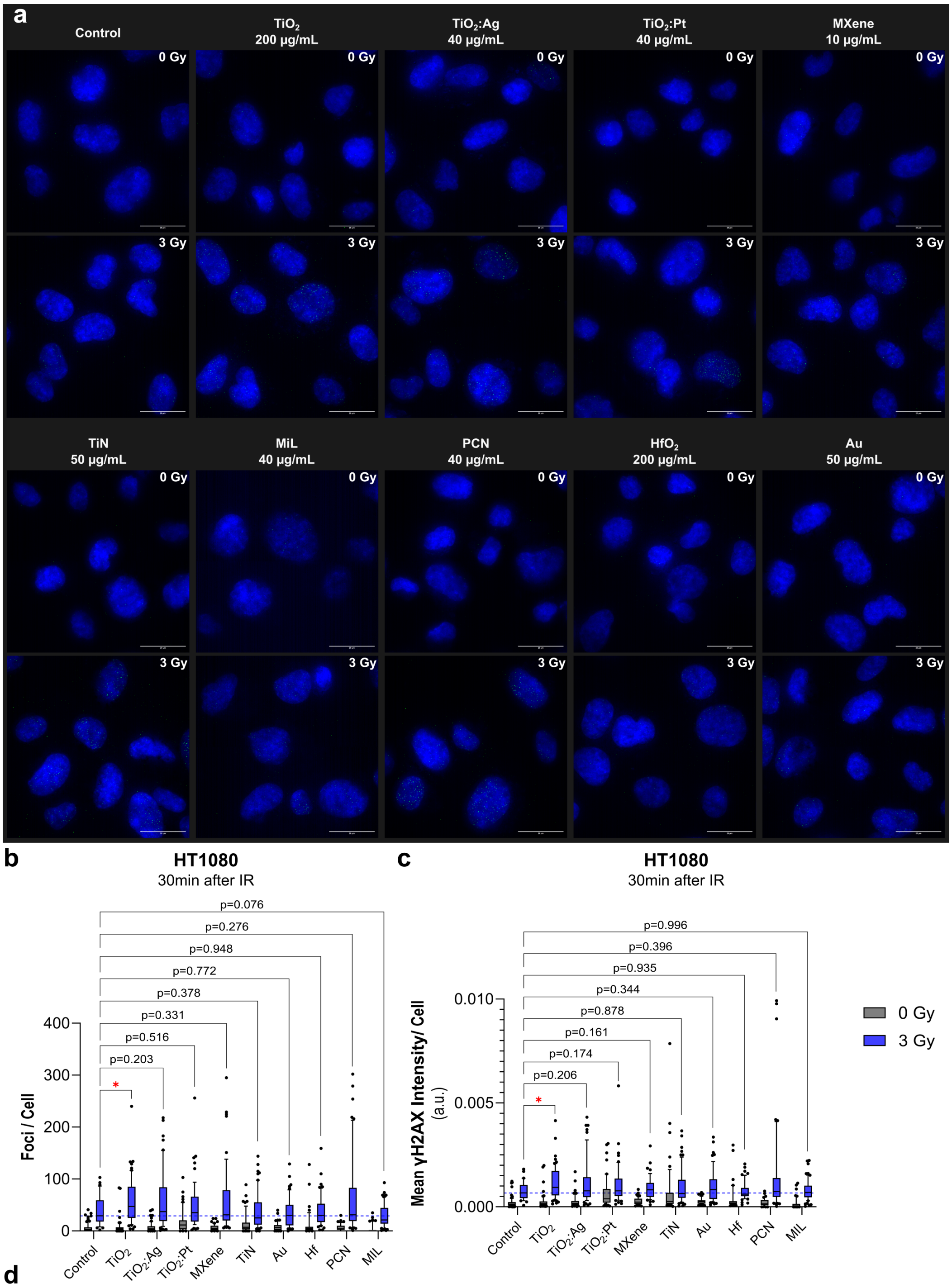

d

Column B	TiO2	TiO2:Ag	TiO2:Pt	MXene	TiN	Au	HfO2	PCN	MIL
vs.	vs.								
Column A	Control								
Foci / Cell: Mann-Whitney *U* test									
P value	0.035	0.203	0.516	0.331	0.378	0.772	0.948	0.276	0.076
Difference of Median	18	8	6	2	-4	1	1	2	-8
Mean γH2AX Intensity / Cell: Mann-Whitney *U* test									
P value	0.016	0.206	0.174	0.161	0.878	0.344	0.935	0.400	0.996
Difference of Median ($x10^3$)	0.269	0.100	0.103	0.151	-0.034	0.157	-0.057	0.078	0.025

Figure S9: Representative immunofluorescence images showing γH2AX foci (green) in nuclei (blue) of HT1080 cells with and without nanomaterials 30 minutes after 0 or 3 Gy irradiation with 6 MV X-rays (a); quantification of foci per nucleus (b) and mean γH2AX intensity per nucleus (c) for 0 and 3 Gy

irradiated cells with and without nanomaterials; the whiskers indicate 10th and 90th percentiles; data points below and above the whiskers are drawn as individual points; boxes extend from the 25th to 75th percentiles; number of analyzed nuclei was around 35 - 65 per condition; statistical results for foci/cell and mean γH2AX intensity per nucleus from the Mann-Whitney U test (d).

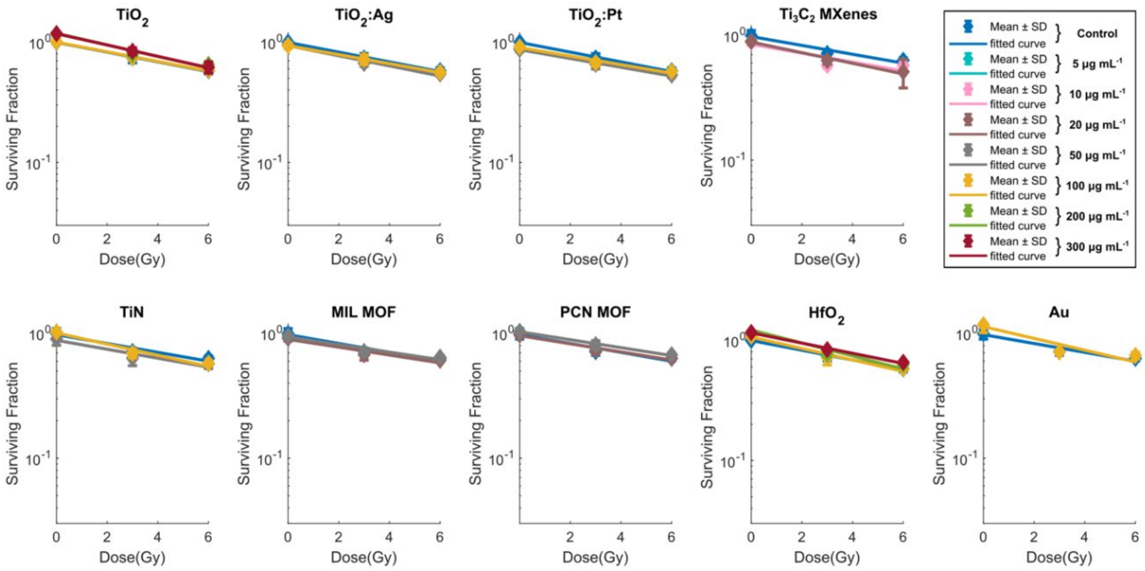

Figure S10: Survival curves (linear quadratic fit) of non-cancerous NHDF cells incubated with different nanomaterials and concentrations and irradiated with 0, 3 and 6 Gy of 6 MV X-rays. The surviving fractions were based on the metabolic activity of the cells 7 days post irradiation. Data (mean ± SD, n = 3) was normalized to the nanomaterial-free control cell viability without irradiation; N = 1.

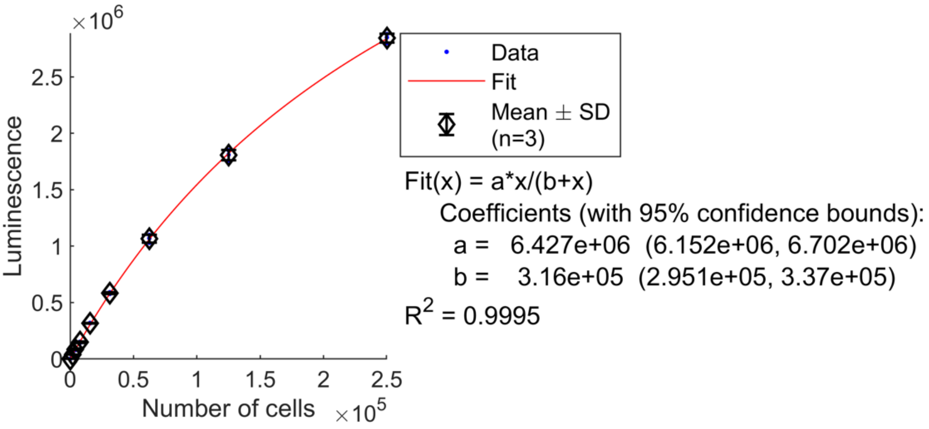

Figure S11: HT1080 cell standard curve to translate luminescence into the number of cells. Cells were seeded in 250 µL cell growth medium and equilibrated for 1h at standard growth conditions (37°C, 5%CO_2). Luminescence was recorded after performing the Cell Titer Glo® assay and a sigmoidal saturation curve was fitted to the data given as mean ± SD (N = 1).
