# Supplementary material for: Catalytically Active Ti‐Based Nanomaterials for Hydroxyl Radical Mediated Clinical X‐Ray Enhancement
Source: Adv Sci (Weinh). 2024 Nov 5;11(47):2406198. doi: 10.1002/advs.202406198 (PMC11653640; doi:10.1002/advs.202406198)
Supplement: Supplementary file 2 — Supporting Information [file ADVS-11-2406198-s002.pdf]

ADVANCED SCIENCE
Open Access

Supporting Information

for *Adv. Sci.*, DOI 10.1002/advs.202406198

Catalytically Active Ti-Based Nanomaterials for Hydroxyl Radical Mediated Clinical X-Ray Enhancement

*Lukas R. H. Gerken, Claire Beckers, Beatrice A. Brugger, Vera M. Kissling, Alexander Gogos, Shianlin Wee, Maria R. Lukatskaya, Hans Schiefer, Ludwig Plasswilm, Martin Pruschy and Inge K. Herrmann**

SUPPLEMENTARY INFORMATION
CellProfiler PIPELINE DOCUMENTATION
CellProfiler Version 4.2.6

Catalytically active Ti-based nanomaterials for hydroxyl radical mediated clinical X-ray enhancement

Lukas R.H. Gerken,[1,2] Claire Beckers,[3] Beatrice A. Brugger,[2] Vera M. Kissling,[2] Alexander Gogos,[1,2] Shianlin Wee,[4] Maria R. Lukatskaya,[4] Hans Schiefer,[5] Ludwig Plasswilm,[5,6] Martin Pruschy[3] and Inge K. Herrmann[1,2,7,8,]*

[1] Nanoparticle Systems Engineering Laboratory, Institute of Energy and Process Engineering (IEPE), Department of Mechanical and Process Engineering (D-MAVT), ETH Zurich, Sonneggstrasse 3, 8092 Zurich, Switzerland.

[2] Particles-Biology Interactions Laboratory, Department of Materials Meet Life, Swiss Federal Laboratories for Materials Science and Technology (Empa), Lerchenfeldstrasse 5, 9014 St. Gallen, Switzerland.

[3] Laboratory for Applied Radiobiology, Department of Radiation Oncology, University Hospital Zurich, University of Zurich, Winterthurerstrasse 190, 8057 Zurich, Switzerland.

[4] Electrochemical Energy Systems Laboratory, Institute of Energy and Process Engineering (IEPE), Department of Mechanical and Process Engineering (D-MAVT), ETH Zurich, Sonneggstrasse 3, 8092 Zurich, Switzerland.

[5] Department of Radiation Oncology, Cantonal Hospital St. Gallen (KSSG), Rorschacherstrasse 95, CH-9007 St. Gallen, Switzerland.

[6] Department of Radiation Oncology, Inselspital University Hospital, 3010 Bern, Switzerland

[7] The Ingenuity Lab, Balgrist University Hospital, Forchstrasse 340, 8008 Zurich, Switzerland.

[8] Faculty of Medicine, University of Zurich, Rämistrasse 71, 8006 Zurich, Switzerland.

*inge.herrmann@empa.ch; ingeh@ethz.ch; +41 (0)58 765 7153

Description of modules used for the CellProfiler analysis:

Corresponding foci stained (green) and nuclei stained (blue) images were loaded into Cell profiler and analyzed pairwise. A total of 15 modules were used for analysis (**Figure 1**). First, the **IdentifyPrimaryObjects** module (**Figure 2**) was used to identify the nuclei from the nuclear stain image using advanced settings, a typical object diameter of min 120 and max 10000 pixel units, a global, two-class Otsu thresholding method with a smoothing scale of 1.3488, a thresholding factor of 1.0, lower and upper bounds on threshold of 0.0 and 1.0, respectively. A by shape method distinguishing clumped objects and drawing dividing lines was used, and the declumping smoothing filter, as well as the minimum allowed distance between local maxima was set to automatic. Holes were filled after both thresholding and declumping. Nuclei Objects were converted to Image and saved using the **ConvertObjectsToImage** (**Figure 3**) and **SaveImages** modules (**Figure 4**). To identify foci in each cell nucleus, foci speckles were enhanced using **EnhanceOrSuppressFeatures** module (**Figure 5**) with a speckle feature size of 10 and a fast speed and accuracy. The enhanced foci image was then masked using the nuclei objects via the **MaskImage** module (**Figure 6**). From the masked foci image each foci was then identified via per-object thresholding using the **IdentifyPrimaryObjects** module (**Figure 7**). The min and max foci diameter were set to 7 and 60 pixel units, respectively. Again, a global, two classes, Otsu thresholding method with a smoothing scale of 1.3488, a thresholding correction factor of 1.0, lower and upper bounds on threshold of 0.2 and 1.0, respectively, was used. A by intensity method distinguishing clumped objects and drawing dividing lines was used with the declumping smoothing filter and the local maxima suppression disitance set to 4. Holes were filled after both thresholding and declumping. Images were saved using **SaveImages** (**Figure 8**) before the nuclei object size was calculated with the **MeasureObjectSizeShape** module (**Figure 9**). Afterwards, the **MeasureObjectIntensity** module (**Figure 10**) was used to measure the intensity of the foci from the original input image per nuclei object. The **RelateObjects** module (**Figure 11**) was used to establish a parent-child between the foci and the nuclei in order to determine which foci (child object) belong to which nuclei (parent object), and to calculate the mean foci measurements for each nucleus. For convenience, the **DisplayDataOnImage** module (**Figure 12**) was used to display the object number of each identified nucleus on the original nucleus input image. The **OverlayOutlines** module (**Figure 13**) was used to verify the foci outlines that have been identified and analyzed. The data displays and foci overlays were saved separately using **SaveImages** (**Figure 14** and **Figure 15**). The measurements made for the nuclei and foci objects was exported with the **ExportToSpreadsheet** module (**Figure 16**).

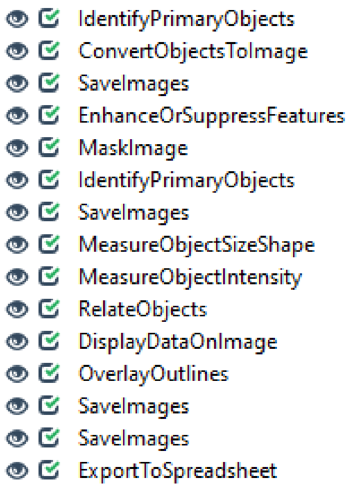

Figure 1: Sequence of all 15 modules used for the CellProfiler analysis.

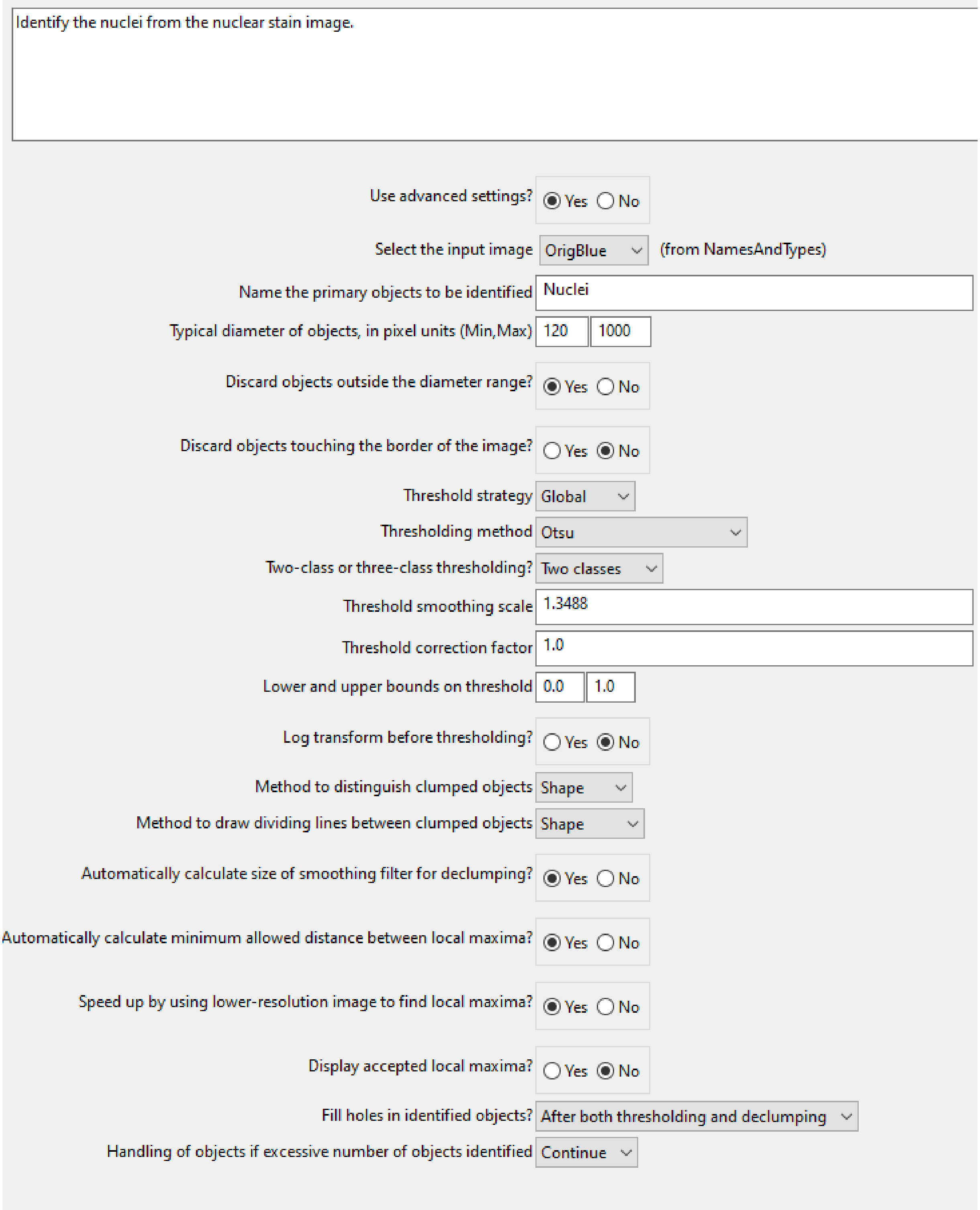

Figure 2: Documentation Module 01 IdentifyPrimaryObjects

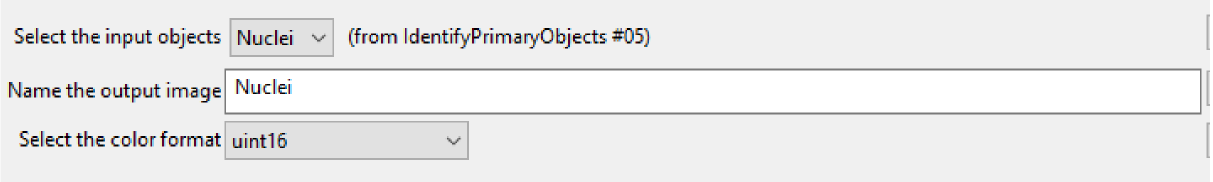

Figure 3: Documentation Module 02 ConvertObjectsToImage

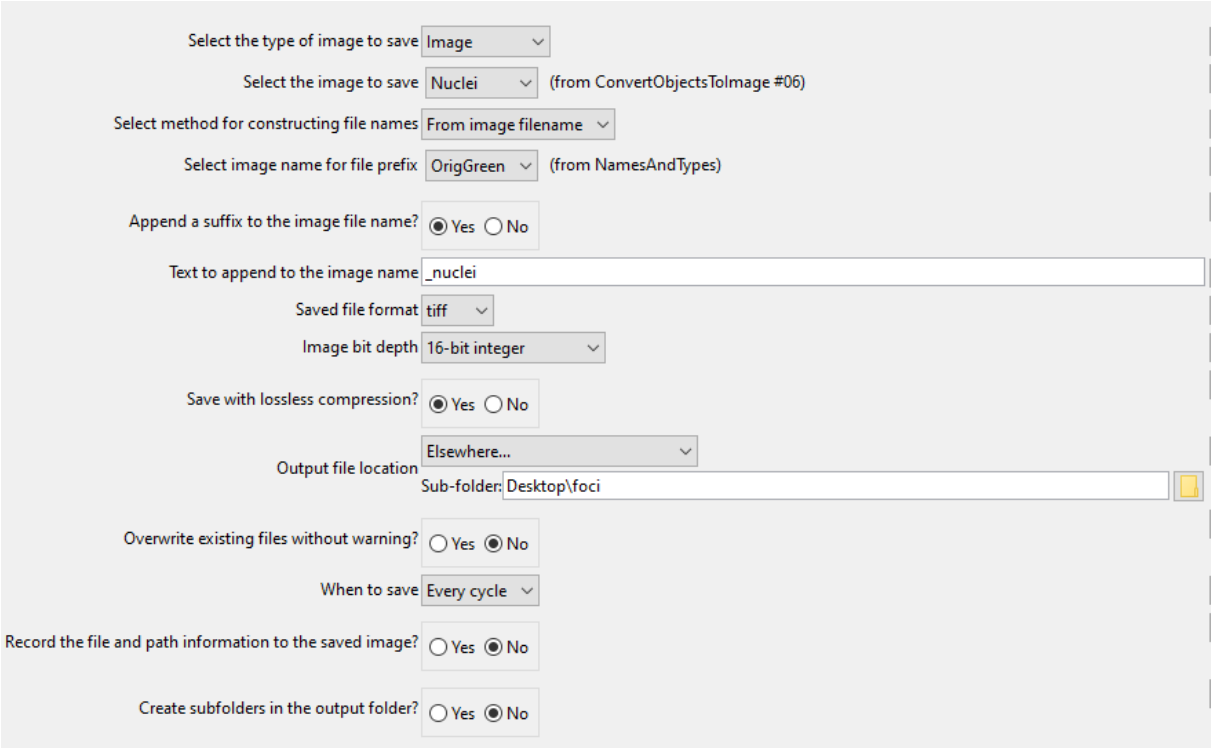

Figure 4: Documentation Module 03 SaveImages

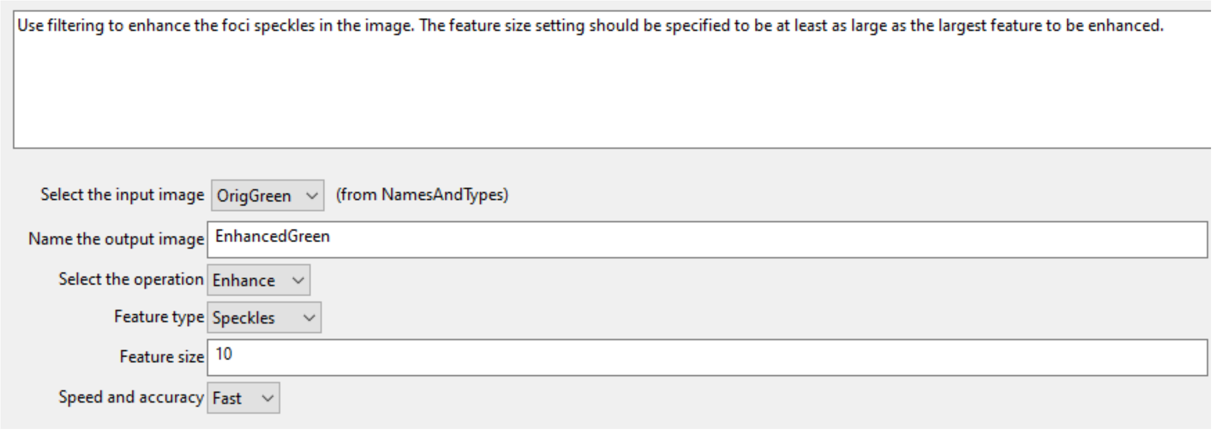

Figure 5: Documentation Module 04 EnhanceOrSuppressFeatures

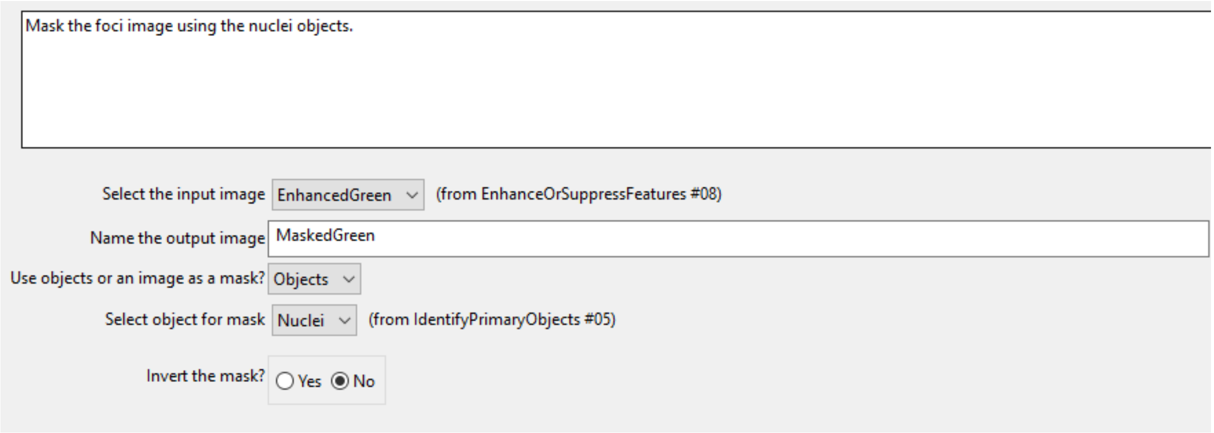

Figure 6: Documentation Module 05 MaskImage

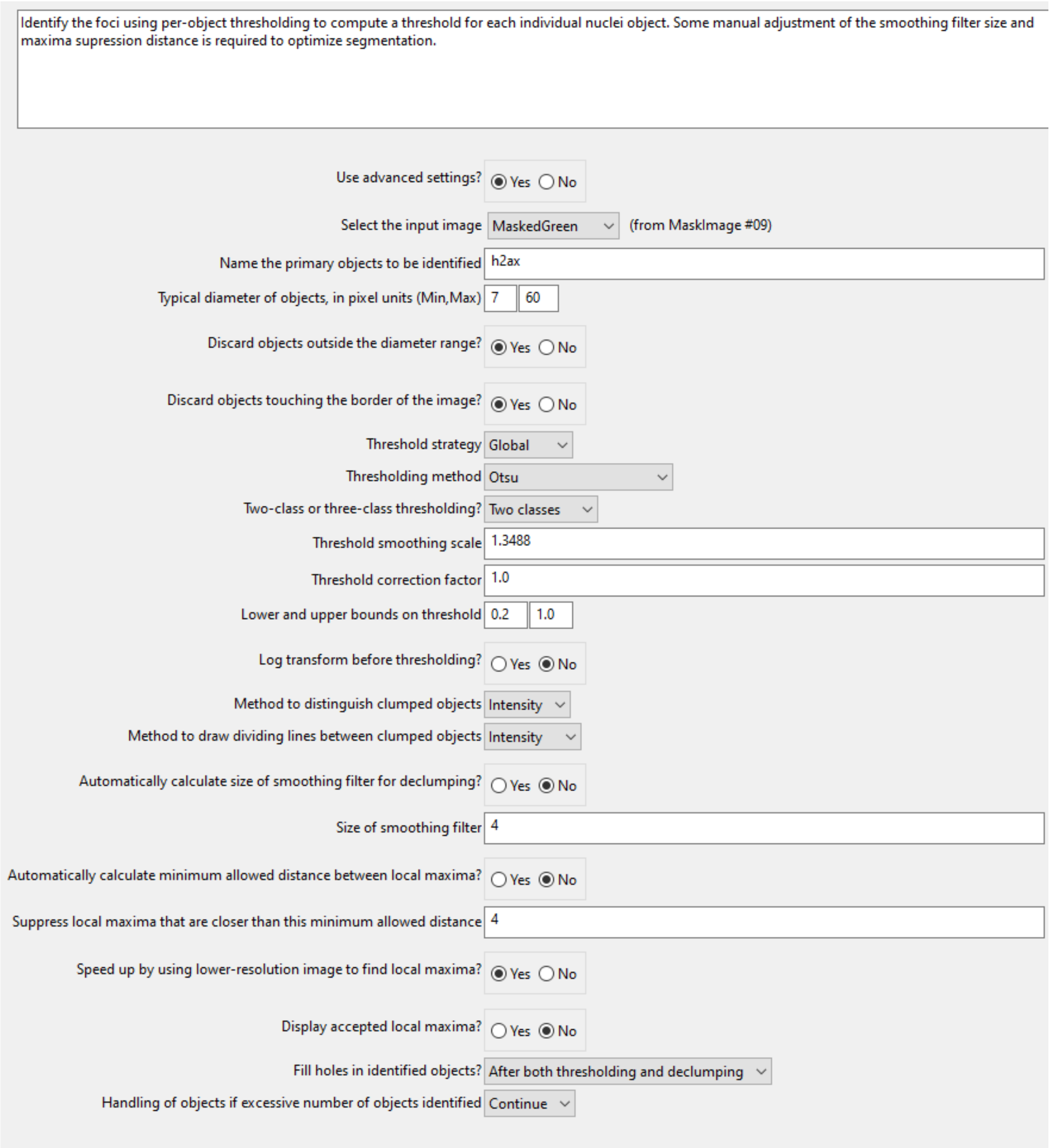

Figure 7: Documentation Module 06 IdentifyPrimaryObjects

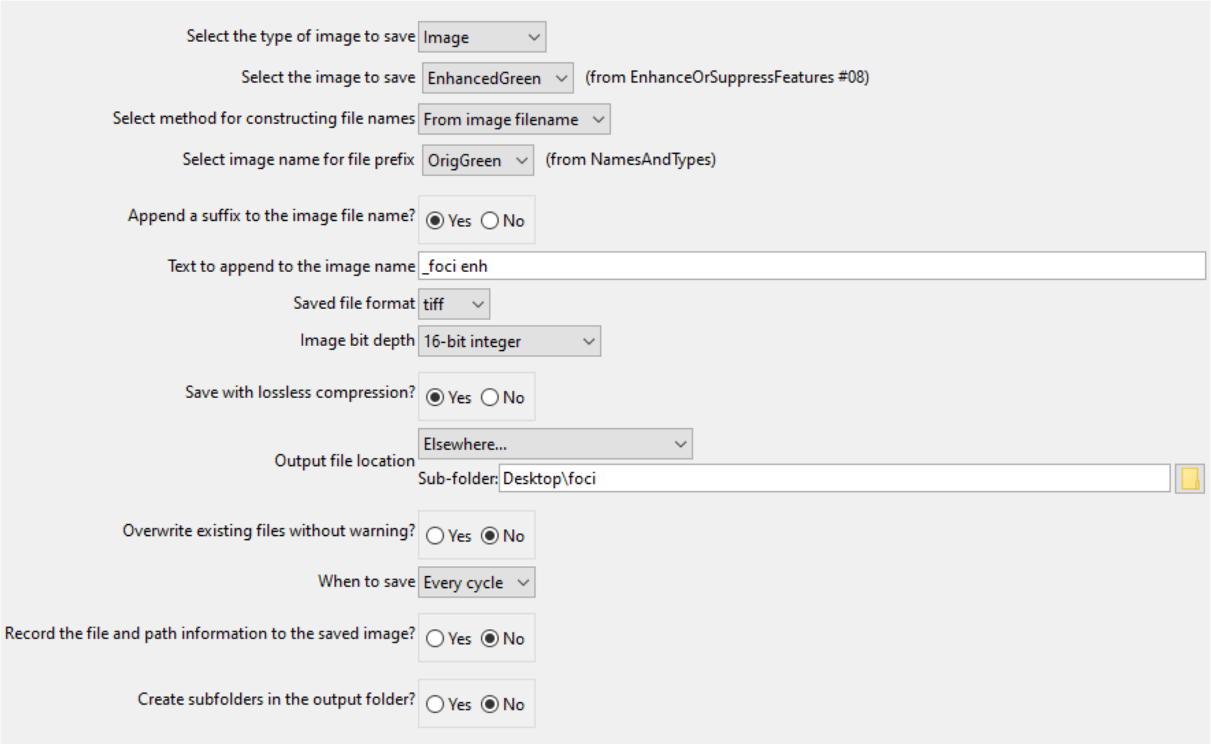

Figure 8: Documentation Module 07 SaveImages

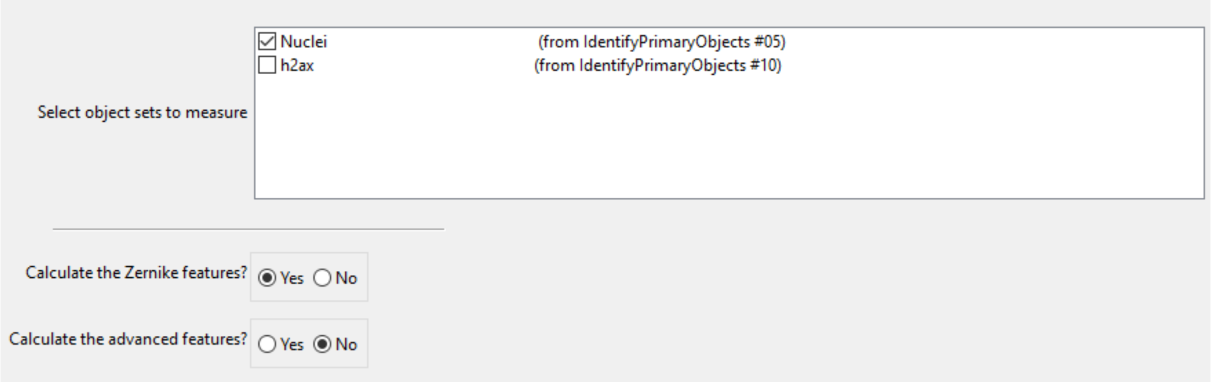

Figure 9: Documentation Module 08 MeasureObjectSizeShape

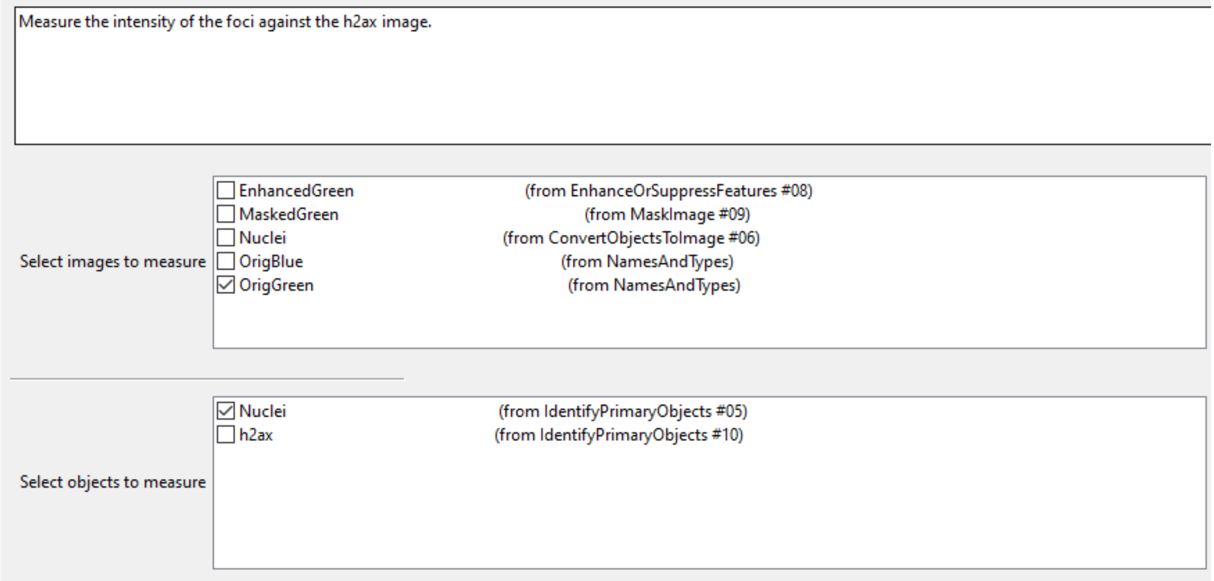

Figure 10: Documentation Module 09 MeasureObjectIntensity

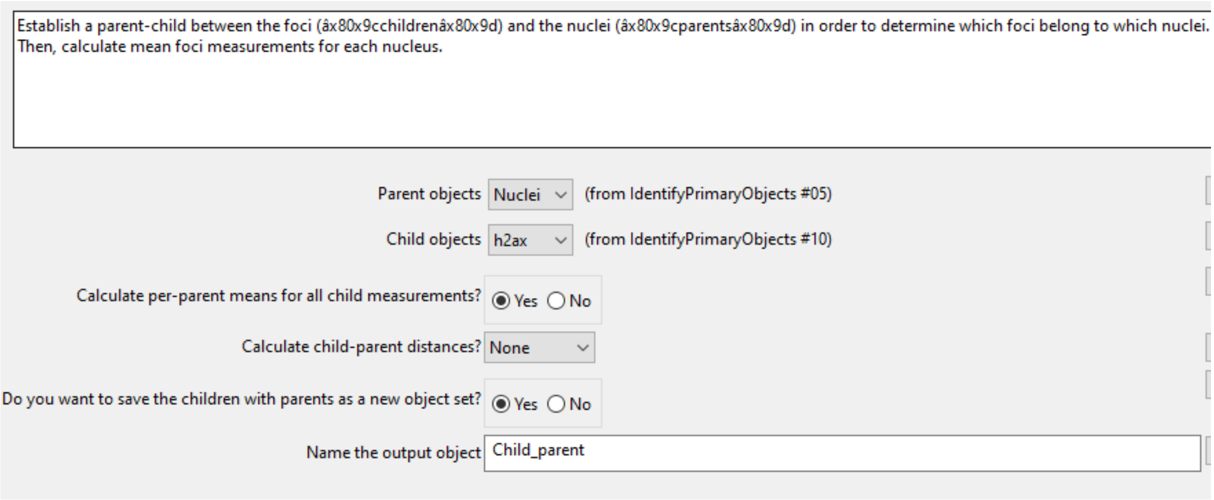

Figure 11: Documentation Module 10 RelateObjects

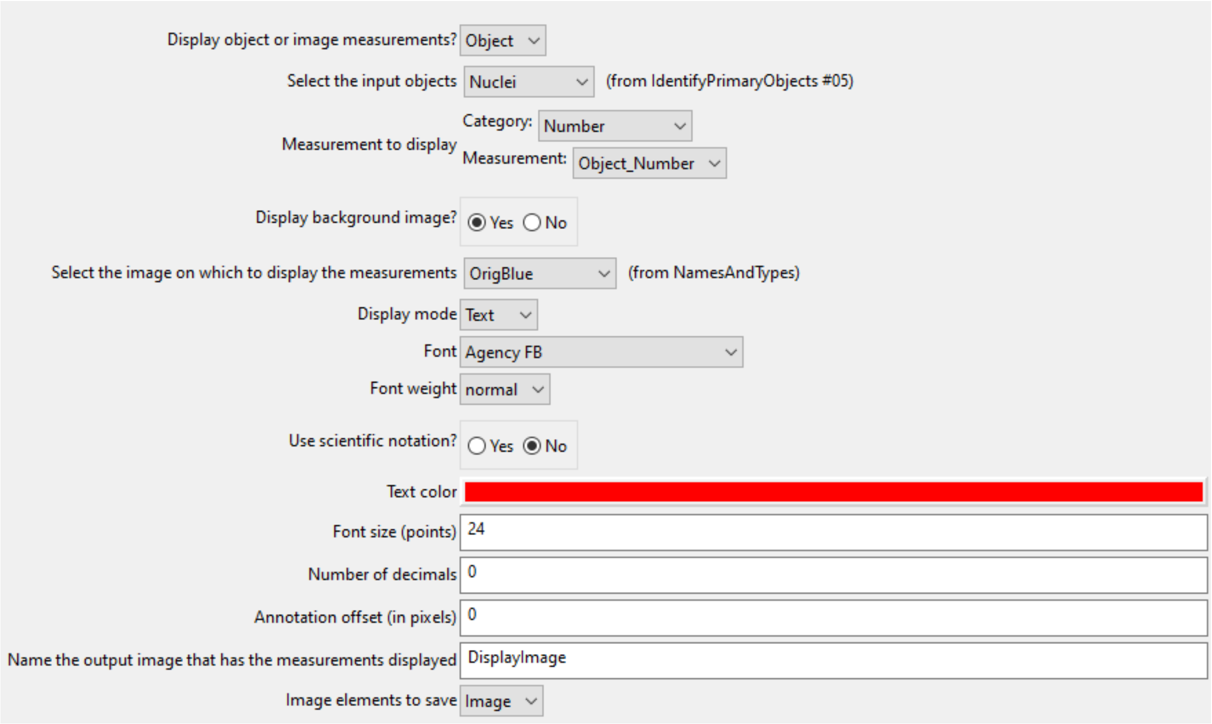

Figure 12: Documentation Module 11 DisplayDataOnImage

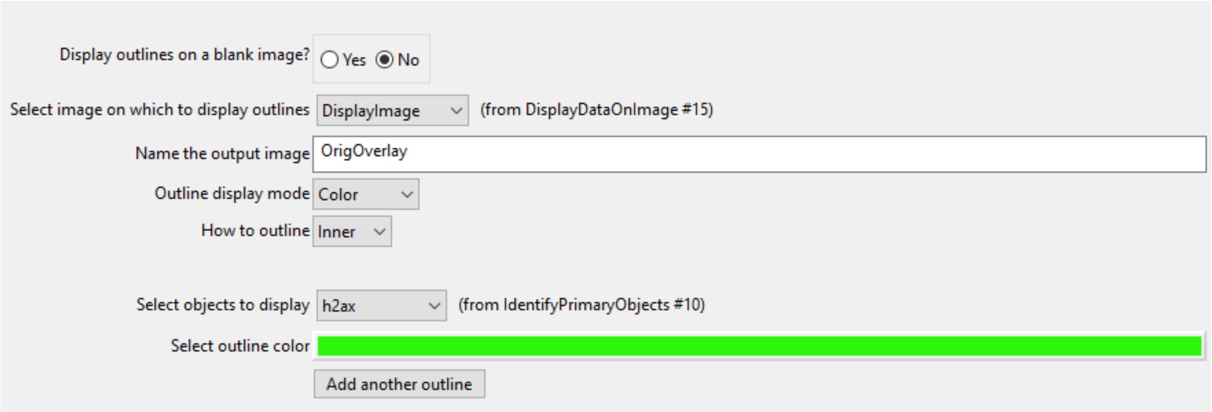

Figure 13: Documentation Module 12 OverlayOutlines

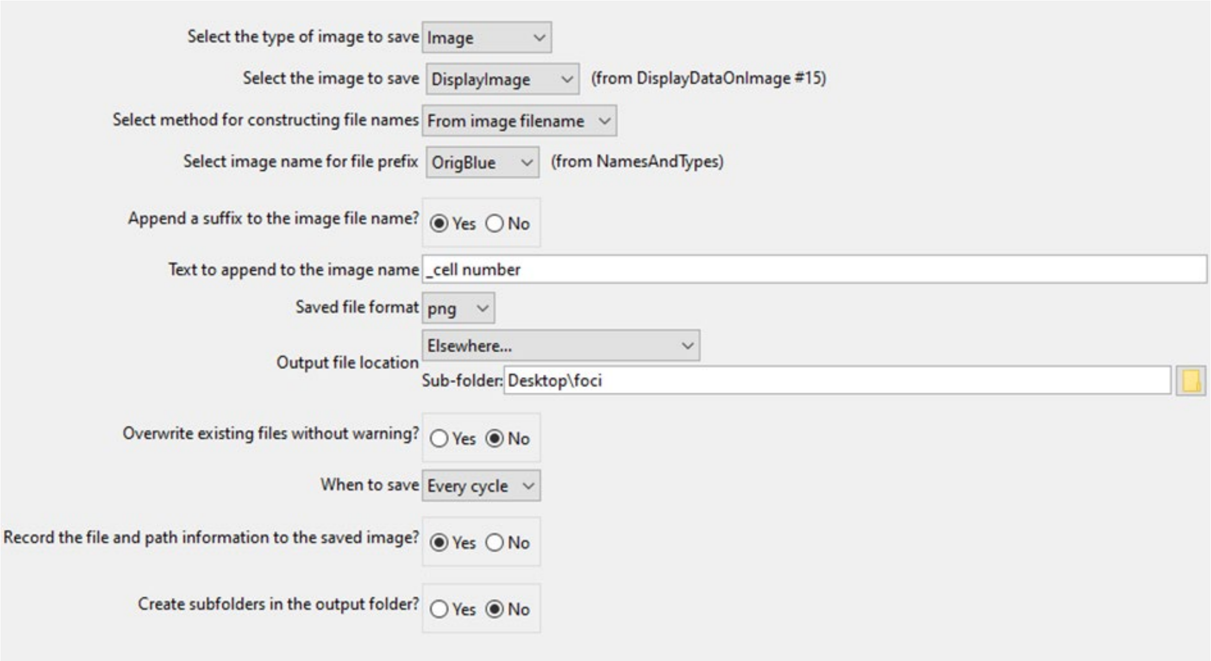

Figure 14: Documentation Module 13 SaveImages

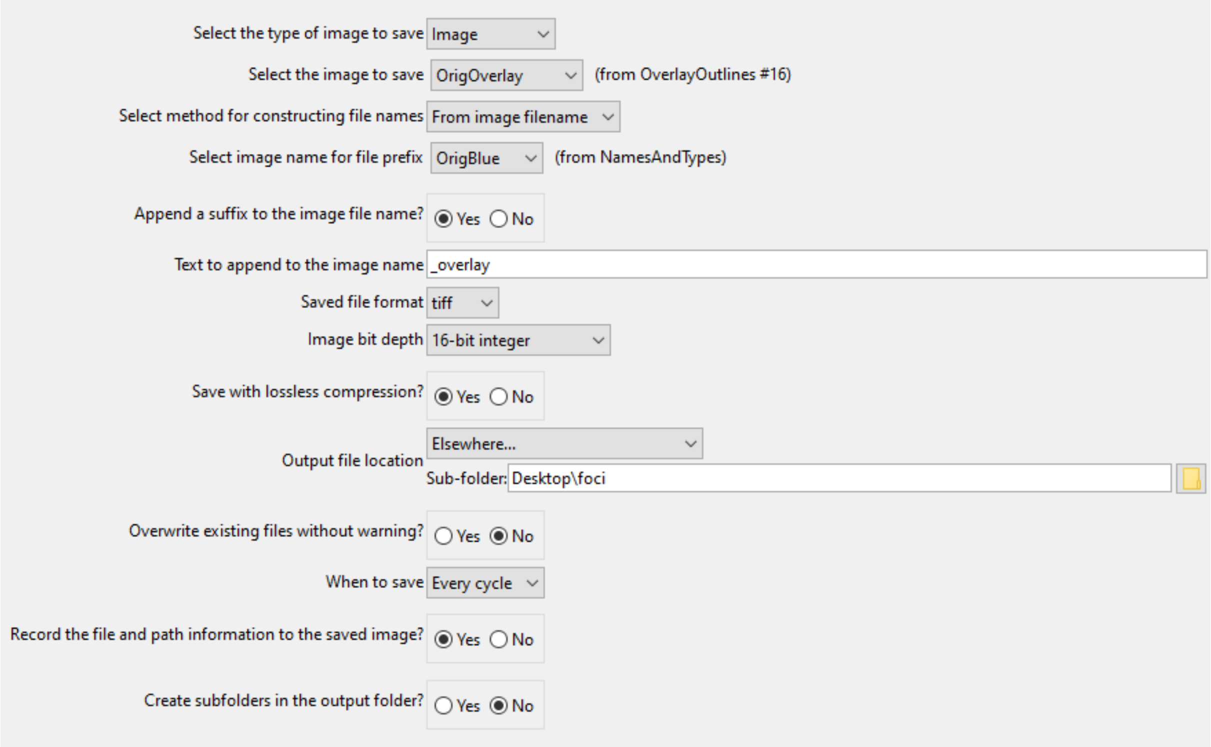

Figure 15: Documentation Module 14 SaveImages

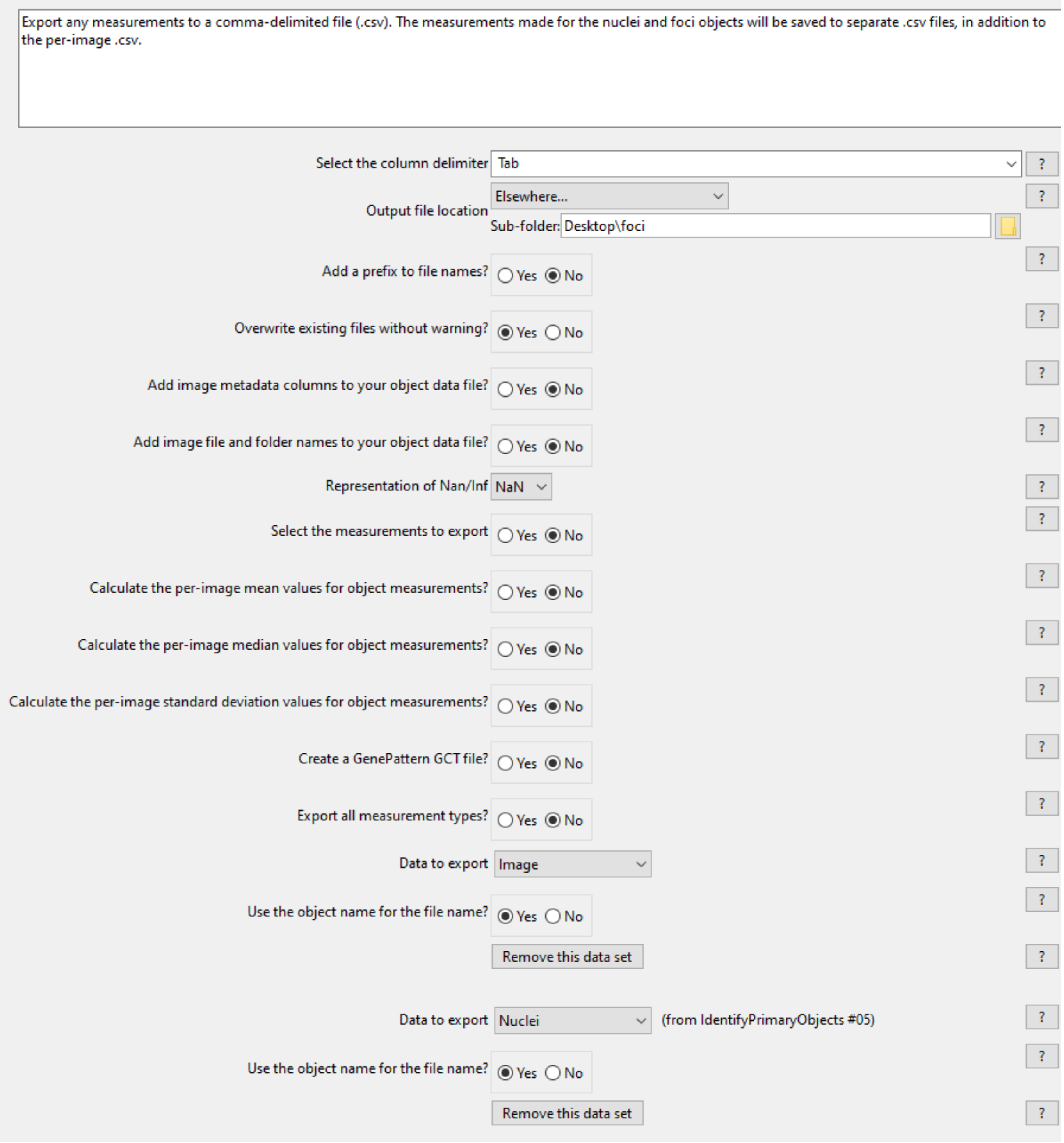

Figure 16: Documentation Module 15 ExportToSpreadsheet
